# Investigating the mechanical stability of flexible metal–organic frameworks

**DOI:** 10.1038/s42004-023-00981-8

**Published:** 2023-09-05

**Authors:** Florencia A. Son, Kira M. Fahy, Madeleine A. Gaidimas, Courtney S. Smoljan, Megan C. Wasson, Omar K. Farha

**Affiliations:** 1 Department of Chemistry and International Institute for Nanotechnology, Northwestern University, Evanston, IL 60208 USA; 2 Department of Chemical and Biological Engineering, Northwestern University, Evanston, IL 60208 USA

**Keywords:** Metal-organic frameworks, Characterization and analytical techniques, Porous materials, Mechanical properties

## Abstract

As we continue to develop metal–organic frameworks (MOFs) for potential industrial applications, it becomes increasingly imperative to understand their mechanical stability. Notably, amongst flexible MOFs, structure-property relationships regarding their compressibility under pressure remain unclear. In this work, we conducted in situ variable pressure powder X-ray diffraction (PXRD) measurements up to moderate pressures (<1 GPa) using a synchrotron source on two families of flexible MOFs: (i) NU-1400 and NU-1401, and (ii) MIL-88B, MIL-88B-(CH_3_)_2_, and MIL-88B-(CH_3_)_4_. In this project scope, we found a positive correlation between bulk moduli and degree of flexibility, where increased rigidity (e.g., smaller swelling or breathing amplitude) arising from steric hindrance was deleterious, and observed reversibility in the unit cell compression of these MOFs. This study serves as a primer for the community to begin to untangle the factors that engender flexible frameworks with mechanical resilience.

## all

Understanding the mechanical properties of metal–organic frameworks (MOFs) is crucial for their industrial application, but the compressibility of structurally dynamic MOFs under pressure remains unclear. Here, in situ variable pressure powder X-ray diffraction experiments on two families of dynamic MOFs find that increased structural flexibility results in enhanced mechanical resilience.

## Introduction

Metal–organic frameworks (MOFs)—porous, crystalline materials composed of inorganic nodes and organic linkers^1^—can be engineered to access a vast library of architectures with potential applications ranging from gas/vapor storage^2–4^, gas/vapor separation^5–7^, and catalysis^8,9^. Regardless of the targeted industrial process for a MOF, some form of post-synthetic processing is required to transform the crystalline powders that are typically synthesized into more viable forms. Shape engineering methods such as pelletization, extrusion, and granulation that facilitate the integration of powders into industrial systems generally require the application of pressure on the MOF crystallites^10–12^. Therefore, it is critical to understand the response of these porous materials to mechanical stress to successfully implement them in the aforementioned applications.

Flexible MOFs, a subdivision of MOFs that reversibly change their form upon exposure to external stimuli (e.g., guest molecules or temperature), have garnered attention over the past decade owing to their responsive structures^13^. While these reversible structural transformations (e.g., breathing, swelling, linker rotation, subnetwork displacement) are desirable for gas sorption applications^14–17^, pressure on the frameworks can also serve as a stimulus that impacts the MOFs’ textural properties, and as such, their efficacy in separations. Moreover, as these pliable materials possess higher degrees of freedom in their movement, further studies must be performed to derive structure-property relationships and understand what factors impact their mechanical stability.

**Fig. 1:**
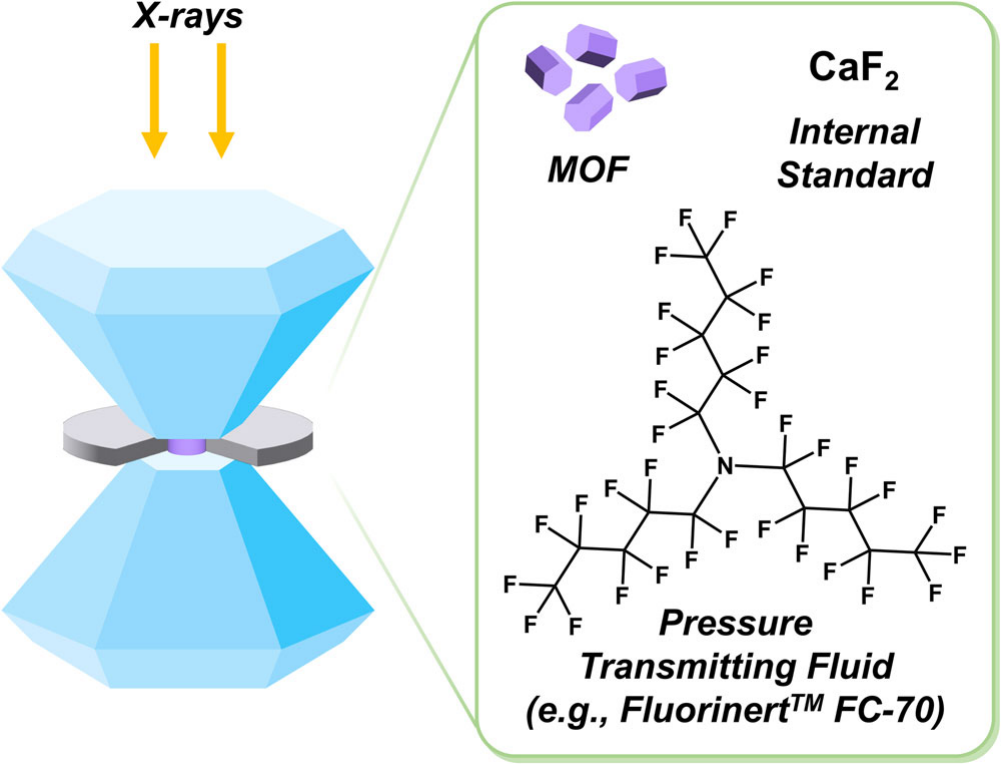
Diagram of a DAC setup, including the sample (MOF), an internal standard, and a non-penetrating pressure transmitting fluid. Gasket cut-out included for viewing purposes only.

Generally, experiments concerned with elucidating the mechanical stability of materials are conducted with advanced techniques such as synchrotron radiation and a diamond anvil cell (DAC) (Fig. 1). Initial work demonstrated how the selection of a pressure transmitting medium (PTM), which converts the uniaxial pressure applied by the DAC into uniform hydrostatic pressure on the sample, influenced the mechanical response of the material. Using fluids that penetrated the pores of the MOF, such as ethanol and/or methanol, decreased compressibility (i.e., increased the bulk moduli) compared to when using non-penetrating PTM, such as Fluorinert^TM^ or mineral oil^18–20^. Therefore, using non-penetrating media provides us with clearer insights into the intrinsic high-pressure response of MOFs.

Preliminary studies probing the mechanical properties of MOFs have mainly focused on more “rigid” scaffolds. Within the Farha group, we have previously performed a systematic study on two topological families of **fcu** and **scu**, and found that the bulk modulus (*K*), a measure of a material’s resistance to hydrostatic compression, of a MOF increases with decreasing void space and linker distortion^21^. Work exploring the node and linker bond for UiO-66(M) (M=Zr, Hf, Ce) indicated that coordination strength plays a role in compressibility, with the softer Ce-carboxylate coordination leading to more facile compression and shearing deformation^22^. Additional studies found strategies to enhance the mechanical stability of a material such as framework catenation^23^ and increasing node connectivity *via* the incorporation of structural linkers^24,25^. When looking at the role of defects in UiO-66(Zr), the bulk modulus of the MOF generally decreased with increasing defects, until the most defective MOF was tested (28.3%) which did not follow trends in defect concentration^26^. Shifting gears to flexible MOFs, researchers found that flexible frameworks can exhibit negative linear compressibility in both experimental^27,28^ and computational efforts^29^ as well as negative area compressibility^30^. Further insights can be found summarized in review articles^31–36^. Despite the research that has been conducted thus far, there still remains a paucity of information regarding trends in the mechanical stability of flexible MOFs.

**Fig. 2:**
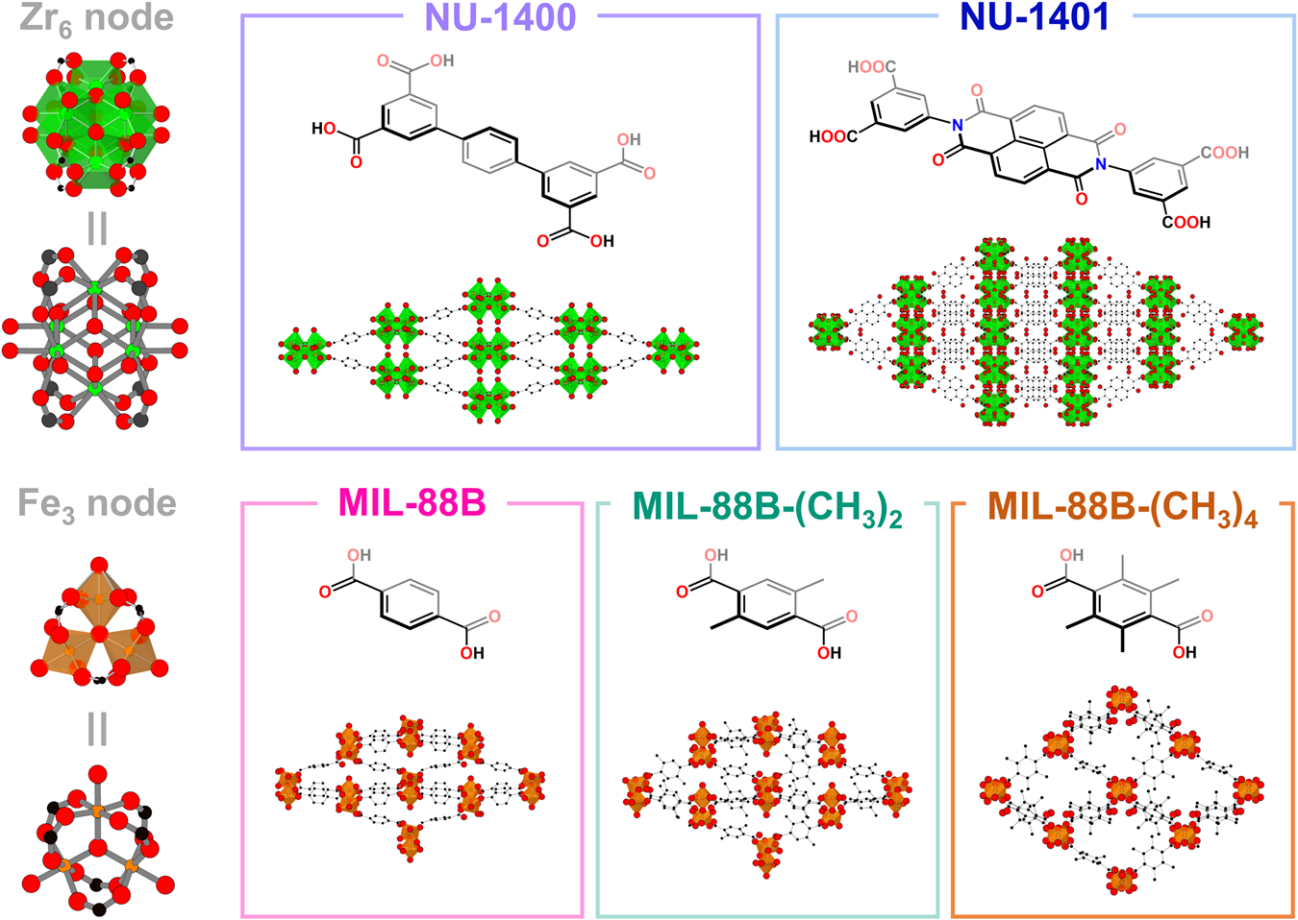
Flexible MOFs in this study with their corresponding nodes, linkers, and structures. Structures are portrayed along the *a*-axis for all MOFs, except for NU-1401 which is depicted along the *b*-axis. Black = carbon; red = oxygen; blue = nitrogen; green = zirconium; orange = iron.

Therefore, herein we report a study on two families of flexible MOFs: (i) NU-1400 and NU-1401, and (ii) MIL-88B, MIL-88B-(CH_3_)_2_, and MIL-88B-(CH_3_)_4_ (Fig. 2). For this work, we consider more flexible MOFs to be those with greater swelling and/or breathing amplitudes upon the inclusion of guest molecules (i.e., larger percentage differences in the unit cell volumes of the open and closed forms of the frameworks). As such, we define more “rigid” MOFs as those that have smaller amplitudes of guest-dependent reversible structural transformations. Through in situ variable powder X-ray diffraction (PXRD) pressure measurements conducted using a synchrotron source at the Advanced Photon Source at Argonne National Laboratory, we investigated structural changes up to moderate pressures (<1 GPa) that are used in post-synthetic processing techniques^37–41^. Interestingly, we found in both systems that increasing rigidity of these MOFs resulted in decreased bulk moduli. Despite having low node connectivity (4-c), NU-1400 and NU-1401 were found to have bulk moduli of 28.1 GPa and 20.0 GPa, respectively. Upon return to ambient conditions after a pressurization campaign, all MOFs reverted back to their initial structures to some degree, indicating reversibility of the structural deformation. While these results provide preliminary insights into the pressure response of flexible materials, we hope that they will encourage others in the community to continue to delineate the variables that affect the mechanical stability of flexible MOFs.

## Results and discussion

To explore the compressibility and mechanical stability of flexible frameworks, we chose two series of MOFs to study. First, we selected NU-1400 and NU-1401 (Fig. 2), which are MOFs with 4-connected Zr_6_ nodes, **lvt** topology, and guest-dependent structural flexibility^42,43^. NU-1400, which is in the *Imma* space group, contracts up to 48% in unit cell volume, depending on the guest molecule present within its pores with expansion along its *b*-axis and contraction along its *c*-axis, as shown through single crystal X-ray diffraction (SCXRD) measurements^42^. Its interpenetrated analog, NU-1401 in the *Ibam* space group, contracts up to 16%, with an increase along its *c*-axis and a decrease in the *a*-axis, also observed through SCXRD studies. Owing to its interpenetrated structure, NU-1401 is less flexible, or has a smaller breathing amplitude, compared to NU-1400. The next set of MOFs examined were a series of MIL-88B^44,45^ analogs that vary in swelling amplitudes—136% for MIL-88B, 96% for MIL-88B-(CH_3_)_2_, and 25% for MIL-88B-(CH_3_)_4_ when determined through PXRD experiments—due to the addition of functional groups on the linker^46^. These trivalent iron-based MOFs with trimeric secondary units are in the *P-62c* space group and decrease in flexibility with an increasing number of methyl groups on the linker (Fig. 2). Prior to the synchrotron experiments, we confirmed the crystallinity of the samples by collecting PXRD patterns under ambient conditions (Supplementary Fig. 1), which agree with the simulated structures of the narrow/closed pore forms of the flexible MOFs. Deviations in peak positions (i.e., larger unit cell parameters than the fully closed forms) are attributed to residual solvent (e.g., higher boiling point solvents such as *N,N*-dimethylformamide) or physisorbed water in the pores of the MOFs. The PXRD pattern of MIL-88B-(CH_3_)_2_ resembles a combination of its narrow and large pore phases^46^. Moreover, we confirmed particle morphologies using scanning electron microscopy (Supplementary Figs. 2–6), which provides further corroboration of the MOFs’ identities. By collecting TGA curves of our samples in the air (Supplementary Fig. 7), we quantified the defects present (Supplementary Table 1) and confirmed the presence of water and residual solvent in the pores. NU-1400, NU-1401, and MIL-88B all possessed missing linker defects. In contrast, MIL-88B-(CH_3_)_2_ and MIL-88B-(CH_3_)_4_ had higher linker/node ratios than their respective ideal values. As the MOFs did not possess a broad peak at low 2θ in their PXRD patterns, which is typically indicative of missing cluster defects, we attribute the greater ratio to be a result of the residual linker that is trapped inside the pores. Further details regarding the characterization of materials can be found in the Supplementary Methods.

**Fig. 3:**
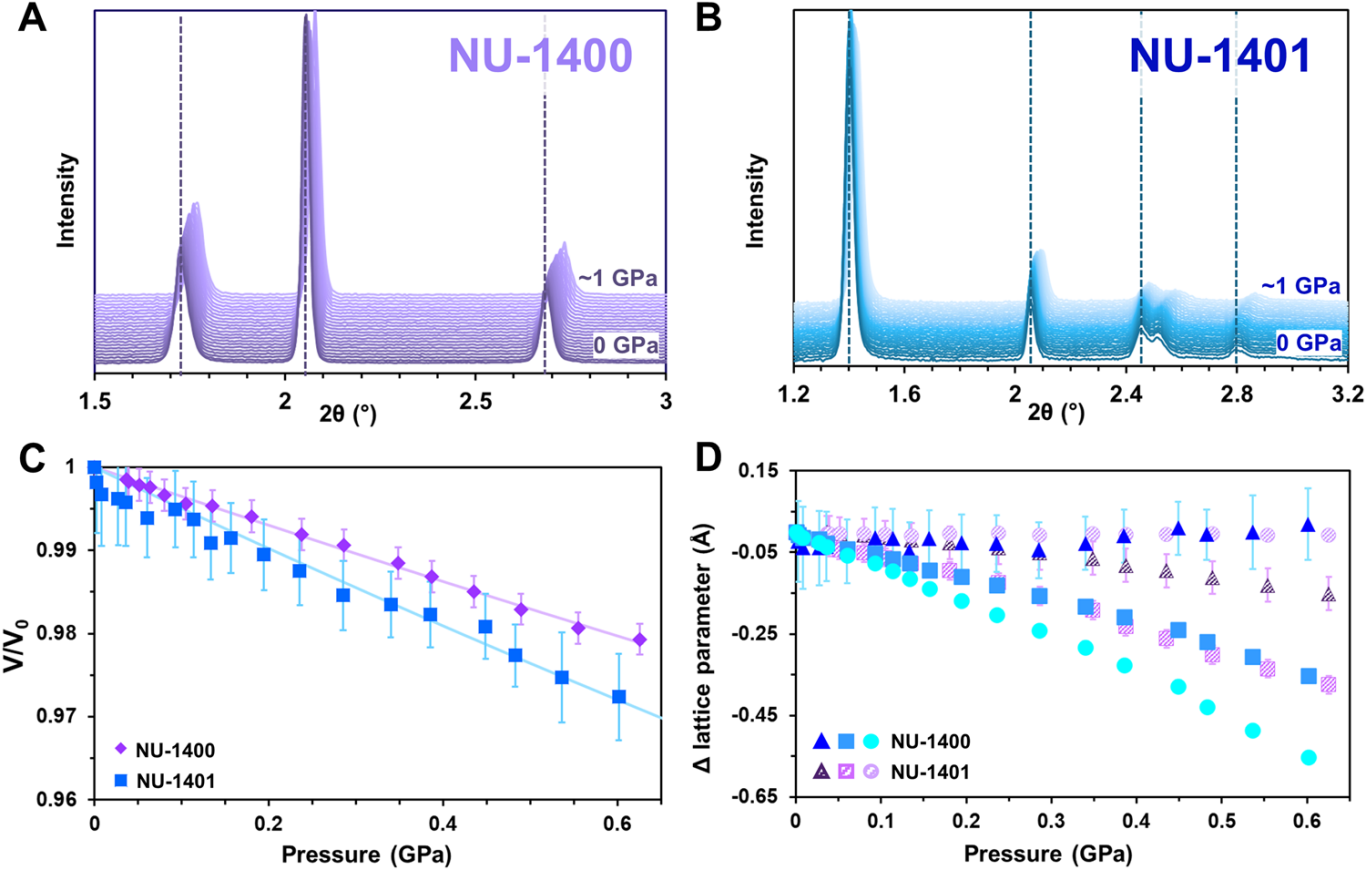
Analysis of NU-1400 and NU-1401. PXRD patterns from 0 to ~1 GPa for **A** NU-1400 (purple) and **B** NU-1401 (blue). **C** P versus *V*/*V*_0_ fit with a 2nd-order Birch-Murnaghan equation of state. **D** Change in lattice parameters with respect to ambient condition measurements, where triangles represent the *a*-axis, squares correspond to the *b*-axis, and circles depict the *c*-axis. Error bars represent estimated standard deviations of fittings.

**Fig. 4:**
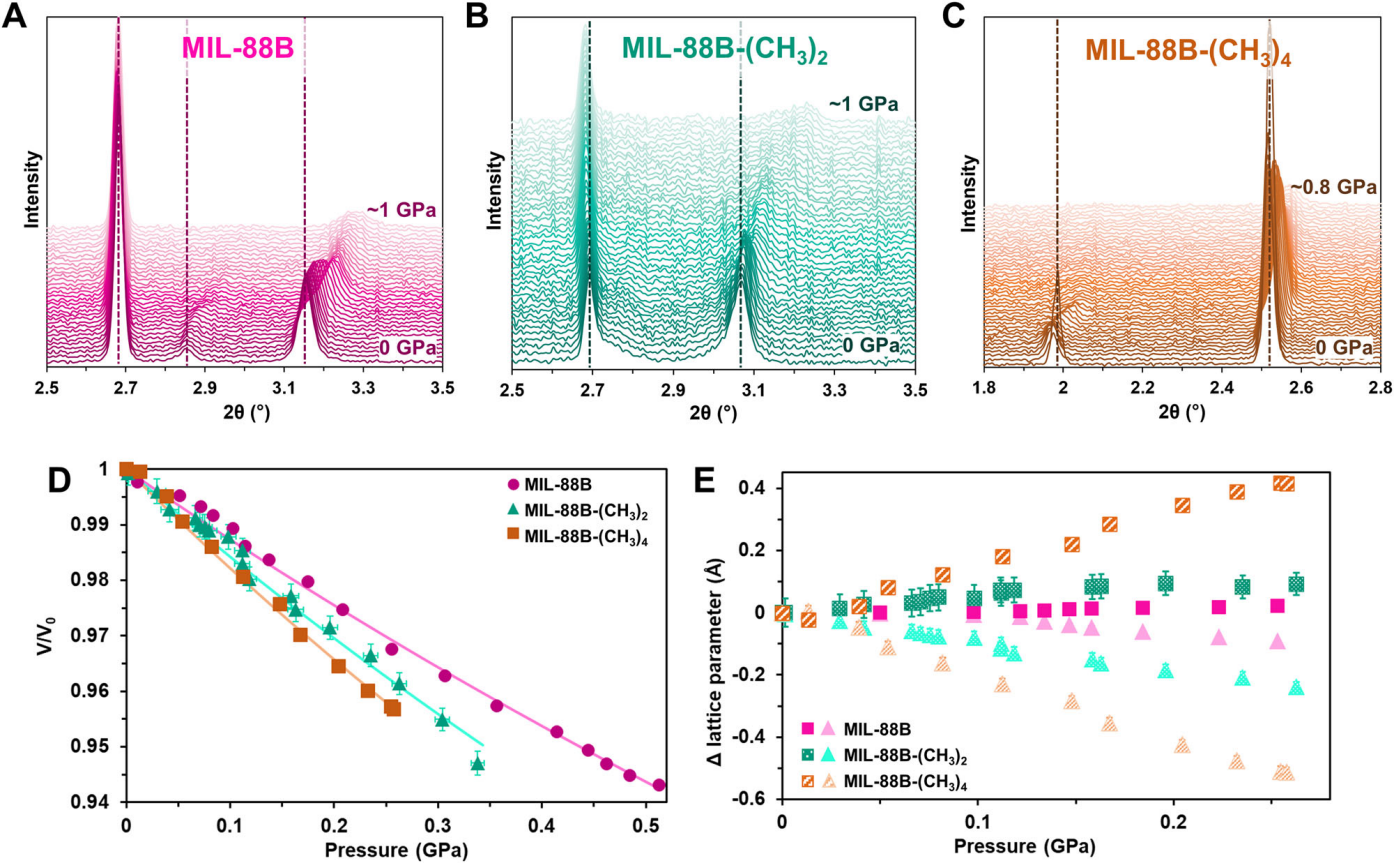
Analysis of MIL-88B, MIL-88B-(CH_3_)_2_, and MIL-88B-(CH_3_)_4_. PXRD patterns from 0 to ~1 GPa for **A** MIL-88B (pink), **B** MIL-88B-(CH_3_)_2_ (green), and **C** MIL-88B-(CH_3_)_4_ (orange). **D** P versus *V*/*V*_0_ fit with a 2nd-order Birch-Murnaghan equation of state. **E** Change in lattice parameters with respect to ambient condition measurements, where squares represent changes in the *c*-axis and triangles represent changes in the *a*-axis. Error bars represent estimated standard deviations of fittings.

We assessed the pressure responses of these selected MOFs using in situ variable pressure PXRD measurements conducted under isothermal conditions at room temperature with a synchrotron source at the 17-BM-B beamline at the Advanced Photon Source, Argonne National Laboratory (Figs. 3 and 4; Supplementary Figs. 13 and 16). Briefly, we packed MOF powders mixed with CaF_2_, used as an internal standard to determine the pressure inside the cell, into indented and drilled stainless-steel gaskets placed inside a membrane-driven diamond anvil cell with 300 μm diameter culet anvils. Then, we added Fluorinert^TM^ FC-70 as the non-penetrating pressure-transmitting fluid. As we had previously shown that FC-70 does not penetrate the channels of NU-1200 (22 Å)^23^, we selected this PTM to analyze the intrinsic pressure response of these microporous MOFs. Pressure campaigns were conducted from 0 to approximately 1 GPa, during which in situ variable pressure PXRD data was collected using monochromatic X-rays (*λ* = 0.45191 Å for NU-1400 and NU-1401; *λ* = 0.45194 Å for MIL-88B, MIL-88B-(CH_3_)_2_, and MIL-88B-(CH_3_)_4_). We processed the raw images using GSAS-II^47^ with calibration data obtained using a LaB_6_ standard and extracted pressure-dependent lattice parameters using Le Bail fits of reported structural models to the diffraction data (Supplementary Figs. 8–12, 14, 15, 17, 18). To obtain the bulk moduli of the samples, isothermal equations of state were fitted to the P versus *V*_0_/*V* data with a 2nd-order Birch-Murnaghan equation of state using EOS-FIT7c and EOS-FIT7-GUI^48,49^. Further details can be found in the methods section.

Using our Le Bail refinements of the NU-1400 and NU-1401 fitted diffraction data (Supplementary Tables 2, 3, 7, and 8), we determined the unit cell parameters of the thermally activated MOFs under ambient conditions, prior to pressurization. Based on these results, NU-1400 had a total unit cell volume of 6806 Å^3^ (*a* = 25.19 Å, *b* = 29.96 Å, *c* = 9.02 Å) and NU-1401 had a volume of 15067 Å^3^ (*a* = 16.25 Å, *b* = 25.15 Å, *c* = 36.86 Å). These values more closely resemble the narrow/closed pore structures, with a thermally activated NU-1400 possessing a volume of 6852 Å^3^^42^_,_ and a supercritically activated NU-1401 having a volume of 15192 Å^3^^43^_._ By extending our analysis to the powder patterns obtained during pressurization, we were able to observe the relationship between unit cell volume and pressure, which could be fit to determine the bulk modulus (*K* = –*V* d*P*/d*V*) of each MOF (Fig. 3, Supplementary Fig. 13). Surprisingly, NU-1400 had a higher bulk modulus (*K* = 28.1 ± 0.3 GPa) than its interpenetrated counterpart NU-1401 (*K* = 20.0 ± 0.3 GPa). These results are a sharp contrast to our prior findings from studying the more rigid NU-1200 (*K* = 5.7 GPa) and STA-26 (*K* = 21.1 GPa)^23^, leading us to believe that the design rules for mechanical stability that have been introduced for more “rigid” frameworks are not fully transferable to flexible ones. Moreover, despite having low node connectivities (4-c), we found that both MOFs are moderately resilient to compression, with NU-1400 approaching the bulk modulus of graphite (*K* = 33.8 GPa)^50^ and UiO-66 (*K* = 37.9 GPa)^21^. Further investigation is required to better understand the mechanical resilience of these MOFs. When looking at the unit cell lattice parameters, there was negligible change in the *c*-axis of NU-1400 and the *a*-axis of NU-1401, while the remaining two parameters for both MOFs decreased with increasing pressure (Fig. 3). The structural transformations upon the inclusion of guest molecules include expansion along the *b*-axis and contraction along the *c*-axis for NU-1400^42^ and expansion along the *c*-axis and contraction along the *a*-axis for NU-1401^43^. Deviations in lattice parameter transformations may arise due to our measurements on MOFs that are in their narrow pore phases.

Parallel analyses on MIL-88B, MIL-88B-(CH_3_)_2_, and MIL-88B-(CH_3_)_4_ revealed ambient condition unit cell volumes of 1833 Å^3^ for MIL-88B (*a* = 10.47 Å, *c* = 19.30 Å), 1986 Å^3^ for MIL-88B-(CH_3_)_2_ (*a* = 10.95 Å, *c* = 19.14 Å), and 3290 Å^3^ for MIL-88B-(CH_3_)_4_ (*a* = 15.13 Å, *c* = 16.60 Å). MIL-88B and MIL-88B-(CH_3_)_2_ more closely resembled their narrow pore volumes of 1485 Å^3^ and 1790 Å^3^, respectively, whereas MIL-88B-(CH_3_)_4_ resembled the open pore volume of ~3500 Å^3^ more than its narrow pore volume of 2810 Å^3^^46^. By fitting the pressure versus unit cell volume data (Supplementary Tables 4, 5, 6, 9, and 10) using a 2nd-order Birch-Murnaghan equation of state (Fig. 4, Supplementary Fig. 16), we found the bulk moduli of the series to be 7.7 ± 0.2 GPa for MIL-88B, 6.1 ± 0.1 GPa for MIL-88B-(CH_3_)_2_, and 5.3 ± 0.1 GPa for MIL-88B-(CH_3_)_4_. All three MOFs expanded along the *c*-axis and contracted along the *a*-axis, with MIL-88B-(CH_3_)_4_ exhibiting the most significant shifts (Fig. 4D), which mirrors the swelling behavior of these MOFs^46^.

In both series of MOFs that we surveyed, the bulk modulus decreases with increasing rigidity (Table 1). Since these materials were assessed in their narrow pore phases, they did not display rapid compression upon pressurization. We postulate that NU-1401 has a lower bulk modulus than NU-1400 due to the steric hindrance imparted by the presence of the interpenetrated network. Both MOFs are flexible owing to a hinge-like motion of the joints between the node and linkers^42^. In NU-1401, however, there is the additional movement of two entangled nets in a scissoring fashion^43^, so we hypothesize that the intertwined frameworks result in decreased mechanical stability due to increased stress on the MOF upon distortion. In the case of the MIL-88B series, we also note a decrease in bulk modulus upon the incorporation of more steric hindrance. Literature examples can be found where steric bulk can diminish the bulk modulus of a material, such as with MIL-53(Cr) (*K* = 4.59 GPa) and MIL-53-CH_3_(Cr) (*K* = 3.7 GPa)^51^ as well as Cu_2_-(bdc)_2_dabco (*K* = 14.0 GPa) and Cu_2_-(DB-bdc)_2_dabco (*K* = 13.5 GPa)^52^. In “rigid” MOFs, we observed a trend of decreasing bulk moduli with decreasing coordination strength when comparing UiO-66(Zr) and UiO-66(Hf) to UiO-66(Ce)^22^. This phenomenon has also been observed in flexible frameworks, where altering the Lewis acidity of the metal can affect coordination strength, as described by Pearson’s hard/soft acid/base theory, and the degree of flexibility^17^. In a study of MIL-53 with differing metals, bulk moduli were measured to be 4.28 GPa for MIL-53(Cr), 10.1 GPa for MIL-53(Fe), and 10.7 GPa for MIL-53(Al)^51^, which correlate with increasing coordination strength. However, the same trend does not hold true for MIL-53-NH_2_(In) with a bulk modulus of 10.9 GPa^53^ compared to MIL-53-NH_2_(Al) with *K* = 7.4 GPa^27^, which highlights the necessity of deeper investigations into flexible MOFs. In comparison to the literature, we posit that rigidity in a framework arising from node-linker coordination strength can be beneficial for enhancing the mechanical stability of MOFs. However, when rigidity arises due to steric effects such as from substituent groups on the linker or interpenetration, MOFs are less capable of withstanding strain from pressure and may face bond cleavage and amorphization. Despite having residual linker trapped in their pores, MIL-88B-(CH_3_)_2_ and MIL-88B-(CH_3_)_4_ still had lower bulk moduli, which further alludes to the detrimental effects steric hindrance may have on the mechanical properties of flexible MOFs.

**Table 1:** Summary of the degree of flexibility (swelling amplitude) and bulk modulus of materials.

MOF	Swelling amplitude	Bulk modulus (GPa)
NU-1400	92%^42^	28.1 ± 0.3
NU-1401	19%^43^	20.0 ± 0.3
MIL-88B	136%^46^	7.7 ± 0.1
MIL-88B-(CH_3_)_2_	96%^46^	6.1 ± 0.1
MIL-88B-(CH_3_)_4_	25%^46^	5.3 ± 0.1

Uncertainties correspond to estimated standard deviation. Swelling amplitudes defined as (*V*_open_ – *V*_dry_)/*V*_dry_ were obtained from the literature.

Finally, we probed the reversibility of the structural deformations for both series of MOFs by collecting powder patterns under ambient conditions after the completion of a pressure campaign. We observed that all MOFs reverted back to their initial structures once the pressure of the cell was released (Supplementary Figs. 19 and 20). However, there were variations in the degree of crystallinity that were maintained after exposure to pressure. Qualitatively, NU-1400, and NU-1401 did not experience a loss in crystallinity after pressurization, whereas in the MIL-88B series, increasing the rigidity of the MOFs resulted in more amorphization and loss in long-range order. We hypothesize that the stronger coordination strength of the Zr-based MOFs may have facilitated the resistance to amorphization. Additionally, for the MIL-88B series, the increased rigidity arising from the more steric hindrance of the functionalized linkers may have been detrimental owing to the increased strain on the bonds upon pressurization. In ZIF-8, a sodalite zeolite-type structure, reversible phase transitions were possible when using methanol/ethanol as the PTM^18^, but irreversible amorphization occurred upon using non-penetrating PTM^19^. For other flexible coordination polymers, pressure-induced structural rearrangements were found to be reversible^54–57^, indicating that although reversibility may not be ubiquitous in flexible frameworks, it is feasible. These qualitative insights could serve as an interesting basis for follow-up studies to explore multiple pressure cycles on flexible MOFs.

## Conclusion

In summary, we conducted in situ variable pressure PXRD measurements on NU-1400, NU-1401, MIL-88B, MIL-88B-(CH_3_)_2_, and MIL-88B-(CH_3_)_4_ to begin to understand how flexibility impacts the mechanical stability of flexible frameworks. We found that as rigidity was increased through increased steric hindrance, the bulk moduli of the MOFs decreased. Additionally, increasing flexibility resulted in higher resilience to pressure-induced amorphization, alluding to flexible MOFs’ ability to withstand more strain on the framework compared to their “rigid” counterparts. These results enable us to address the research gaps in the mechanical stability of flexible MOFs by setting preliminary structure-property relationships. As the scope of our study was two series of MOFs, we encourage others in the field to continue exploring the mechanical properties of flexible frameworks concomitantly with complementary techniques such as Raman spectroscopy and/or computational simulations.

## Methods

### MOF syntheses

NU-1400^42^, NU-1401^43^, MIL-88B, MIL-88B-(CH_3_)_2_, and MIL-88B-(CH_3_)_4_ were synthesized according to reported procedures^46^, with a few minor modifications denoted below.

#### Synthesis of NU-1400

In a 100 mL bottle, ZrOCl_2_•8H_2_O (256.8 mg, 0.797 mmol) was dissolved in *N,N*-diethylformamide (24 mL), and formic acid (15 mL) via sonication, then placed in an 80 °C oven for 1 h. After cooling to room temperature, [1,1′:4′,1″]terphenyl-3,3″,5,5″-tetracarboxylic acid (81.6 mg, 0.201 mmol) and deionized water (1.2 mL) were added and sonicated for 20 min. The product was isolated via centrifugation, washed with DMF three times, and acetone three times, soaking for 1 h between each wash. After soaking the MOF overnight in acetone, the product was collected via centrifugation and placed in an 80 °C vacuum oven overnight.

#### Synthesis of NU-1401

*N,N*’-bis(5-isophthalic acid)naphthalenediimide (BINDI) was synthesized according to reported procedures, with a few modifications. Briefly, in a 50 mL round-bottomed flask, 1,4,5,8-naphthalenetetracarboxylic dianhydride (0.810 g, 3 mmol) and aminoisophthalic acid (1.096 g, 6 mmol) were mixed in propionic acid (25 mL). The mixture was placed in an aluminum heating block, fitted with a condenser, and refluxed at 165 °C for 48 h while stirring. After cooling down to room temperature, water was added to precipitate the product, which was then isolated by vacuum filtration. The filtrate was washed with water (500 mL), followed by ethanol (100 mL). The product was purified via recrystallization by placing it in DMF (150 mL) in a 120 °C oven for 30 min and then sonicating until dissolved. After cooling overnight, the product was collected by filtration and then washed with ethanol (750 mL). The formation and purity of the linker were confirmed using ^1^H NMR spectroscopy (Supplementary Fig. 21).

To synthesize the MOF, ZrOCl_2_•8H_2_O (85.6 mg, 0.266 mmol) and BINDI (40 mg, 0.0668 mmol) were mixed with DMF (4.5 mL) and formic acid (2 mL) in a 4-dram vial and sonicated until dissolved. The solution was placed in a 130 °C oven for 48 h. After cooling, the product was collected by centrifugation, washed with DMF three times, and ethanol three times, soaking for 1 h between each wash. The product was soaked overnight in ethanol, collected by centrifugation, and dried overnight in an 80 °C oven.

#### Synthesis of MIL-88B

FeCl_3•_6H_2_O (270 mg, 1 mmol) and 1,4-benzenedicarboxylic acid (116 mg, 1 mmol) was combined with DMF (5 mL) and NaOH (2 M, 0.4 mL) and sonicated until fully dissolved. The mixture was transferred to a Teflon-lined Parr Vessel and placed in a 100 °C oven for 12 h. The MOF was recovered via centrifugation and washed with DMF three times and ethanol 3 times, soaking for 1 h in between each wash. MIL-88B was soaked overnight in ethanol, collected via centrifugation, and then dried overnight in an 80 °C vacuum oven.

#### Synthesis of MIL-88B-(CH_3_)_2_

FeCl_3•_6H_2_O (270 mg, 1 mmol) and 2,5-dimethyl-1,4-benzenedicarboxylic acid (194 mg, 1 mmol) were combined with methanol (5 mL) and sonicated for 5 min. The mixture was transferred to a Teflon-lined Parr Vessel and placed in a 100 °C oven for 3 days. The MOF was recovered via centrifugation and washed with DMF three times and ethanol 3 times, soaking for 1 h in between each wash. MIL-88B-(CH_3_)_2_ was soaked overnight in ethanol, collected via centrifugation, and then dried overnight in an 80 °C vacuum oven.

#### Synthesis of MIL-88B-(CH_3_)_4_

FeCl_3•_6H_2_O (270 mg, 1 mmol) and 2,3,5,6-tetramethyl-1,4-benzenedicarboxylic acid (222 mg, 1 mmol) was combined with DMF (5 mL) and sonicated until fully dissolved. The mixture was transferred to a Teflon-lined Parr Vessel and placed in a 100 °C oven for 3 days. The MOF was recovered via centrifugation and washed with DMF three times and ethanol 3 times, soaking for 1 h in between each wash. MIL-88B-(CH_3_)_2_ was soaked overnight in ethanol, collected via centrifugation, and then dried overnight in an 80 °C vacuum oven.

### In situ variable pressure powder X-ray diffraction measurements

#### Gasket preparation

In all, 250 μm thick stainless-steel gaskets were indented to 100 μm thickness using a membrane-driven diamond anvil cell with 300 μm diameter culet anvils. After, a 250 μm hole was drilled into the center of the indented gasket using a laser micro-machining system^58^. Drilled and indented gaskets were cleaned by sonicating in ethanol.

#### Sample preparation

MOF powders, which were dried overnight in a vacuum oven at 80 °C, were gently mixed with the internal standard CaF_2_ (~20% *v*/*v*) using a mortar and pestle. The 250 μm thick stainless-steel gaskets that were indented to 100 μm thickness and drilled with a 250 μm diameter hole were then placed in the DAC with 300 μm culet anvils. The MOF/CaF_2_ mixture was packed into the hole of the gasket, ensuring no loose powder around the culet’s indent, and then the piston of the DAC was carefully closed back onto the cylinder. After fully closing the cell, without applying pressure to the gasket, an ambient pressure PXRD pattern was collected. The cell was then opened, and Fluorinert^TM^ FC-70 was added to the sample mixture as the non-penetrating pressure-transmitting fluid. After resealing, a compression membrane that is driven by a methanol syringe pump system was fitted onto the DAC. In situ variable pressure PXRD data were collected using monochromatic X-rays at room temperature at the 17-BM-B beamline at the Advanced Photon Source, Argonne National Laboratory in combination with a Varex 4343CT area detector. Powder patterns were collected with 6 s exposures, totaling 1 min per image, as the pressure was increased from 0 to 1 GPa. After the pressure campaign was completed, the pressure was released back to ambient conditions, and a final pattern was measured.

#### Data processing

Raw images were processed using GSAS-II^47^, with calibration data obtained using a LaB_6_ standard. Using GSAS-II, pressure-dependent lattice parameters were extracted using Le Bail fits of reported structural models to the diffraction data. Isothermal equations of state were fit to the P versus *V*_0_/*V* data with a 2nd order Birch-Murnaghan equation of state using EOS-FIT7c and EOS-FIT7-GUI which use a least-squares minimization method of the differences between the observed and calculated pressures^48,49^.

### Supplementary information

Supplementary Information

## Data Availability

Raw diffraction data analyzed in this study are available from authors upon request.
