# Supplementary material for: Investigating the mechanical stability of flexible metal–organic frameworks
Source: Commun Chem. 2023 Sep 5;6:185. doi: 10.1038/s42004-023-00981-8 (PMC10480183; doi:10.1038/s42004-023-00981-8)
Supplement: Supplementary file 1 — Supplementary Information [file 42004_2023_981_MOESM1_ESM.pdf]

Investigating the Mechanical Stability of Flexible Metal–Organic Frameworks

Florencia A. Son[1], Kira M. Fahy[1,†], Madeleine A. Gaidimas[1,†], Courtney S. Smoljan[2,†], Megan C. Wasson[1,†], Omar K. Farha[1,2,*]

[1]Department of Chemistry and International Institute for Nanotechnology, Northwestern University, Evanston, Illinois 60208, United States
[2]Department of Chemical and Biological Engineering, Northwestern University, Evanston, IL 60208, United States of America
[†]Authors contributed equally to this paper
*Corresponding Author: o-farha@northwestern.edu

Table of Contents

I. Supplementary methods S2
II. Ambient pressure powder X-ray diffraction (PXRD) patterns S3
III. Scanning electron microscopy (SEM) images S4–S5
IV. Thermogravimetric analysis (TGA) S6–S7
V. Le Bail fits for in situ variable pressure PXRD trials S8–S25
VI. Reversibility of compression measurements S26
VII. ^{1}H NMR spectrum of linker S27

I. Supplementary methods

Powder X-Ray Diffraction (PXRD): Ambient pressure PXRD measurements were collected on a STOE STADI P with a CuKα1 radiation source (λ = 1.54056 Å) at the IMSERC X-ray Facility at Northwestern University. *In situ* variable pressure PXRD patterns were collected using monochromatic X-rays at the 17-BM-B beamline at the Advanced Photon Source, Argonne National Laboratory in combination with a Varex 4343CT area detector. Additional details can be found in the main text.

Scanning Electron Microscopy (SEM): SEM images were obtained using a FEI Quanta 650 at the EPIC facility (NUANCE Center-Northwestern University). The samples were coated with 18 nm of osmium using an SPF Osmium Coater (NUANCE Center-Northwestern University) prior to imaging.

Thermogravimetric Analysis (TGA): Defects were quantified by collecting weight loss data under air on a Mettler Toledo TGA/DSC 1 Star System instrument. Samples were heated in air from 30 °C to 100 °C at a rate of 10 °C/minute. The samples were then held at 100 °C for 20 minutes to allow for the desorption of residual methanol, acetone, ethanol, and/or water. Following this, the samples were ramped up to 600 °C at a rate of 10 °C/minute. The sample was held at 600 °C for 10 minutes and then cooled back down to 30 °C at a rate of -10 °C/minute. A TGA curve for a blank aluminum pan was collected as a background measurement and subtracted from the sample data.

II. Ambient pressure powder X-ray diffraction (PXRD) patterns

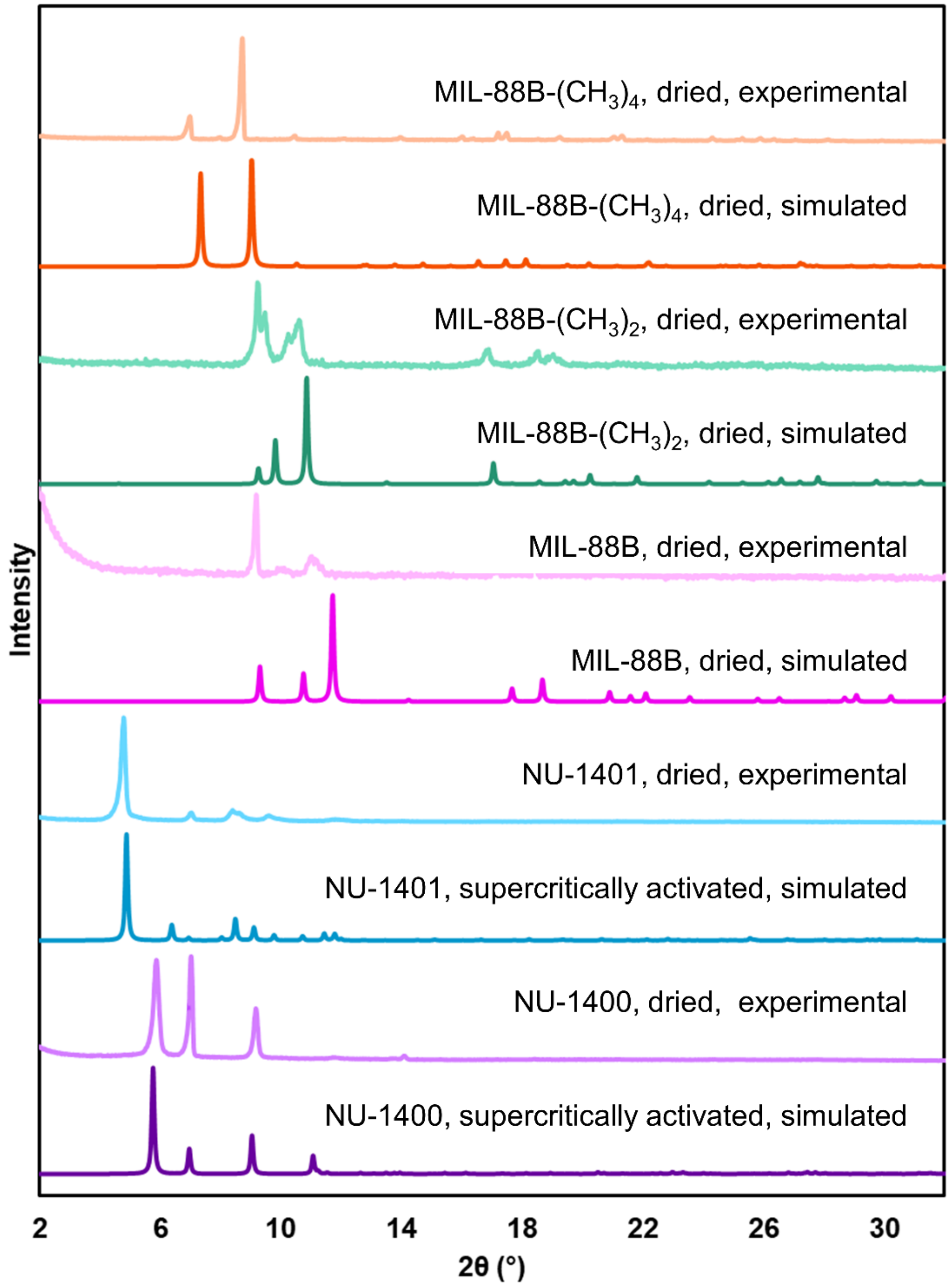

Supplementary Figure 1. Simulated and experimental powder X-ray diffraction patterns of MOFs under ambient conditions (CuKα radiation, λ = 1.54056 Å).

III. Scanning electron microscopy (SEM) images

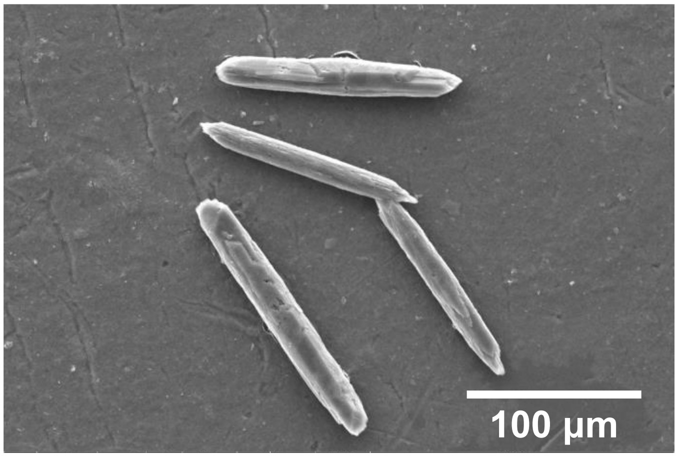

Supplementary Figure 2. SEM of NU-1400

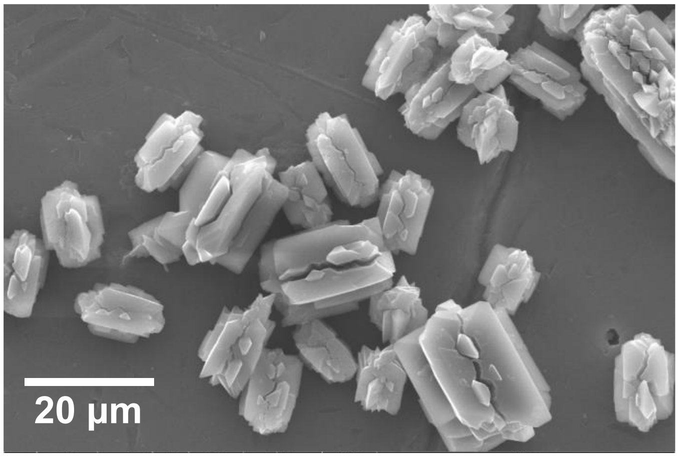

Supplementary Figure 3. SEM of NU-1401

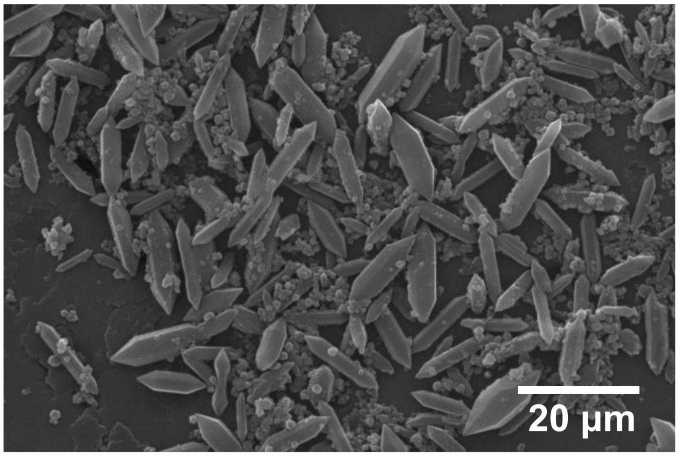

Supplementary Figure 4. SEM of MIL-88B

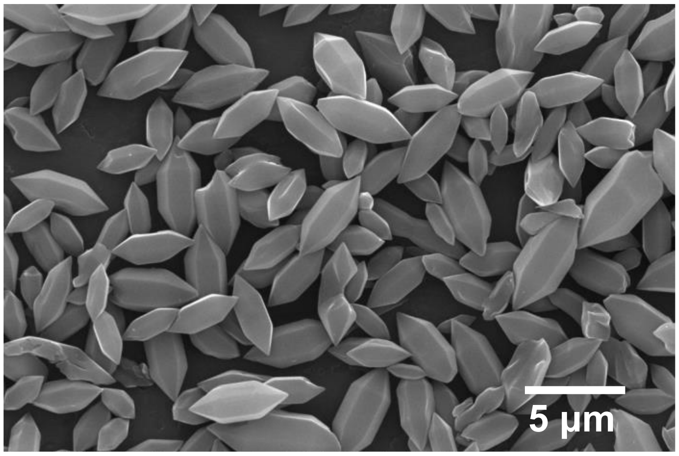

Supplementary Figure 5. SEM of MIL-88B-$(CH_3)_2$

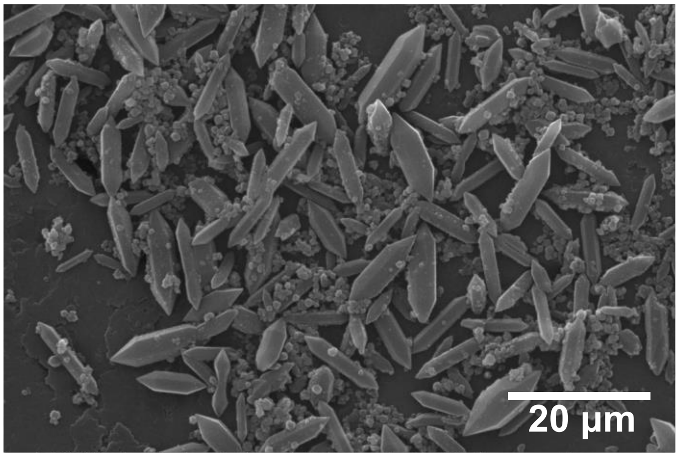

Supplementary Figure 6. SEM of MIL-88B-$(CH_3)_4$

IV. Thermogravimetric analysis

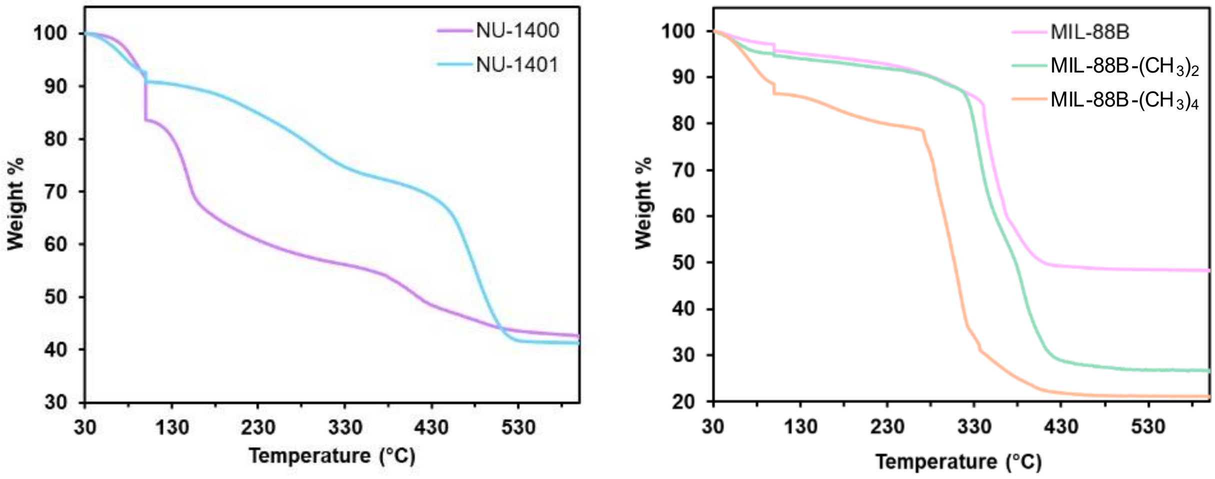

Supplementary Figure 7. Thermogravimetric analysis of samples under air. Initial weight loss from 30 to 100 °C corresponds to loss of water.

Supplementary Table 1. TGA curves of samples for defect analysis. Total weight percent loss of linker was calculated by subtracting the final mass at 600 °C from the initial mass at the specified temperature, and then dividing by the initial mass at the specified temperature. Calculations corresponding to mass lost from linker were conducted at 100 °C for MIL-88B, corresponding to sample with no physisorbed water. Initial masses for MIL-88B-$(CH_3)_2$ and MIL-88B-$(CH_3)_2$ were set to be at 200 °C corresponding to loss of physisorbed DMF. Finally, NU-1400 and NU-1401 calculations were conducted starting from 350 °C to account for loss of solvent in the pores and formate molecules on the node.

Initial temperatures for weight % loss analysis were selected based on the boiling points of the residual solvents present. *N,N*-dimethylformamide (DMF, b.p. = 153 °C), *N,N*-diethylformamide (DEF, b.p. = 178 °C), water (b.p. = 100 °C), ethanol (b.p. = 78 °C), acetone (b.p. = 56 °C). Linkers/node were calculated using the assumption that the residual mass at 600 °C corresponds to a metal oxide (ZrO_2 or Fe_2O_3).

MOF	Residual solvent present	Temperature range for weight loss calculation	Weight % linker	Weight % metal oxide	Experimental linkers/node	Ideal linkers/ node
NU-1400	DEF, water, DMF, ethanol	350–600 °C	24.0	76.0	0.58	1
NU-1401	DMF, ethanol	350–600 °C	43.8	56.2	0.98	1
MIL-88B	water, acetone	100–600 °C	49.7	50.3	1.4	1.5
MIL-88B-$(CH_3)_2$	methanol, DMF, ethanol	200–600 °C	71.3	28.7	3.1	1.5
MIL-88B-$(CH_3)_4$	DMF, ethanol	200–600 °C	74.1	25.9	3.0	1.5

Example calculation for NU-1400 with the assumption of 24.0 g linker and 76.0 g ZrO_2 based on percent weight loss data from TGA curves:

$$24\ g\ linker\ \left(\frac{mol\ TPTC^{4-}\ linker}{402.318\ g}\right) = 0.0595\ moles\ linker$$

$$76\ g\ ZrO_2\ \left(\frac{mol\ ZrO_2}{123.218\ g}\right)\left(\frac{1\ mol\ Zr}{1\ mol\ ZrO_2}\right)\left(\frac{1\ mol\ Zr_6\ node}{6\ mol\ Zr}\right) = 0.103\ moles\ node$$

$$\frac{0.0595\ moles\ linker}{0.103\ moles\ node} = 0.577\ linkers/node$$

NU-1400, NU-1401, and MIL-88B all possess missing linker defects. We attribute the higher linker/node ratios of MIL-88B-$(CH_3)_2$ and MIL-88B-$(CH_3)_4$ to residual linkers trapped inside their pores, rather than missing cluster defects, owing to the lack of broad peaks in the PXRD patterns at low 2θ values that are typically indicative of missing clusters in MOFs.

V. Le Bail fits for *in situ* variable pressure PXRD trials

Supplementary Table 2. Parameters of Le Bail fits to variable pressure PXRD data for NU-1400

**Pressure (GPa)**	**V CaF_2 (Å^3)**	**a NU-1400 (Å)**	**b NU-1400 (Å)**	**c NU-1400 (Å)**	**V NU-1400 (Å^3)**	**Rwp (%)**
0	163.220(5)	25.190(4)	29.961(7)	9.018(6)	6806(6)	18.61
0.037(2)	163.142(3)	25.189(4)	29.931(7)	9.015(7)	6796(6)	20.06
0.039(2)	163.136(4)	25.188(4)	29.924(7)	9.015(7)	6795(6)	20.16
0.051(3)	163.113(4)	25.186(4)	29.916(7)	9.014(7)	6792(6)	20.15
0.064(3)	163.057(3)	25.182(4)	29.911(7)	9.014(7)	6790(5)	20.17
0.080(2)	163.010(3)	25.177(4)	29.899(7)	9.012(7)	6783(5)	20.10
0.104(2)	162.950(3)	25.171(4)	29.885(7)	9.008(7)	6776(6)	20.05
0.135(2)	162.863(3)	25.163(4)	29.866(7)	9.014(7)	6775(5)	20.17
0.180(2)	162.752(3)	25.152(4)	29.838(7)	9.016(7)	6766(5)	20.08
0.238(2)	162.659(3)	25.139(3)	29.804(7)	9.011(7)	6751(5)	19.96
0.286(2)	162.539(3)	25.125(3)	29.769(7)	9.014(7)	6742(5)	19.74
0.348(2)	162.465(3)	25.108(3)	29.729(7)	9.013(7)	6728(5)	19.57
0.387(2)	162.373(3)	25.096(3)	29.699(6)	9.012(7)	6717(5)	19.25
0.435(2)	162.270(3)	25.078(3)	29.660(6)	9.013(7)	6704(5)	19.16
0.489(2)	162.145(4)	25.060(3)	29.626(6)	9.011(7)	6690(5)	19.19
0.555(2)	162.008(4)	25.039(3)	29.587(6)	9.010(7)	6675(5)	18.95
0.625(2)	161.846(5)	25.014(3)	29.543(6)	9.019(7)	6665(5)	19.11
0.711(2)	161.720(6)	24.986(3)	29.492(6)	9.016(7)	6644(5)	19.16
0.775(3)	161.585(6)	24.965(3)	29.448(6)	9.007(7)	6622(5)	19.21
0.846(4)	161.454(7)	24.942(3)	29.404(6)	9.013(7)	6610(5)	19.29
0.916(4)	161.310(9)	24.922(3)	29.362(6)	9.013(7)	6595(5)	19.17

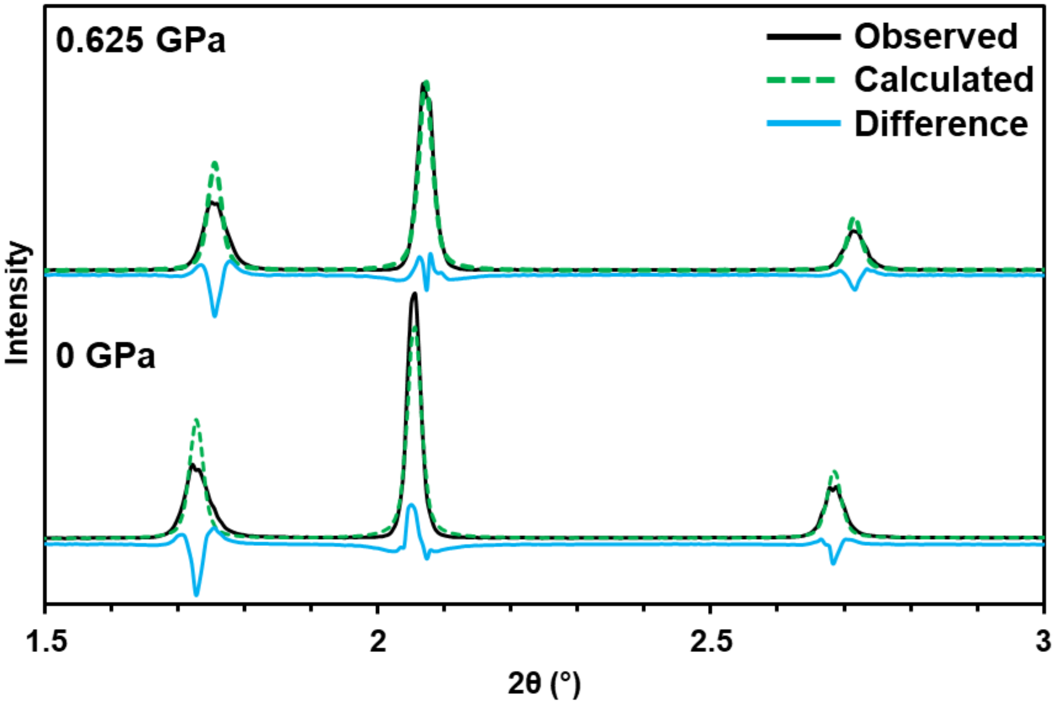

Supplementary Figure 8. Le Bail fittings of NU-1400 at 0 GPa and 0.625 GPa.

Supplementary Table 3. Parameters of Le Bail fits to variable pressure PXRD data for NU-1401

**Pressure (GPa)**	**V CaF_2 (Å^3)**	**a NU-1401 (Å)**	**b NU-1401 (Å)**	**c NU-1401 (Å)**	**V NU-1401 (Å^3)**	**Rwp (%)**
0	163.248(0)	16.25(13)	25.15(12)	36.86(12)	15067(120)	16.16
0.003(4)	163.235(7)	16.23(10)	25.15(13)	36.85(12)	15040(92)	16.55
0.008(3)	163.224(7)	16.21(10)	25.14(13)	36.84(12)	15018(93)	16.36
0.027(4)	163.187(7)	16.21(9)	25.14(13)	36.83(12)	15010(84)	16.54
0.036(3)	163.170(6)	16.22(8)	25.13(13)	36.82(12)	15004(79)	16.84
0.06(4)	163.123(7)	16.20(8)	25.11(13)	36.80(13)	14976(72)	16.85
0.093(3)	163.060(7)	16.24(8)	25.10(13)	36.78(13)	14991(70)	17.04
0.114(3)	163.020(6)	16.23(7)	25.09(13)	36.76(13)	14973(68)	17.24
0.133(3)	162.982(7)	16.20(7)	25.08(13)	36.74(13)	14930(66)	17.36
0.156(3)	162.937(6)	16.24(7)	25.06(13)	36.72(13)	14939(64)	17.53
0.194(3)	162.864(6)	16.22(7)	25.05(14)	36.69(13)	14909(64)	17.84
0.236(3)	162.784(6)	16.22(7)	25.02(14)	36.65(13)	14880(63)	17.99
0.285(2)	162.688(5)	16.21(7)	25.00(14)	36.62(14)	14835(63)	18.26
0.34(3)	162.583(6)	16.22(6)	24.97(14)	36.57(14)	14818(59)	18.18
0.385(3)	162.496(6)	16.24(7)	24.95(14)	36.53(14)	14800(60)	18.14
0.449(3)	162.374(6)	16.26(6)	24.92(15)	36.48(14)	14779(59)	18.57
0.482(3)	162.309(6)	16.25(6)	24.88(14)	36.43(14)	14726(56)	18.29
0.536(4)	162.206(7)	16.25(9)	24.85(14)	36.37(13)	14686(82)	17.23
0.601(4)	162.081(8)	16.27(9)	24.80(14)	36.31(13)	14651(79)	17.22
0.663(4)	161.963(8)	16.26(9)	24.76(14)	36.25(13)	14600(77)	16.84
0.741(5)	161.814(9)	16.29(8)	24.72(14)	36.18(13)	14571(70)	16.65
0.808(7)	161.686(14)	16.32(7)	24.66(14)	36.09(12)	14524(66)	16.24
0.878(8)	161.554(16)	16.31(7)	24.62(14)	36.03(12)	14469(63)	15.85

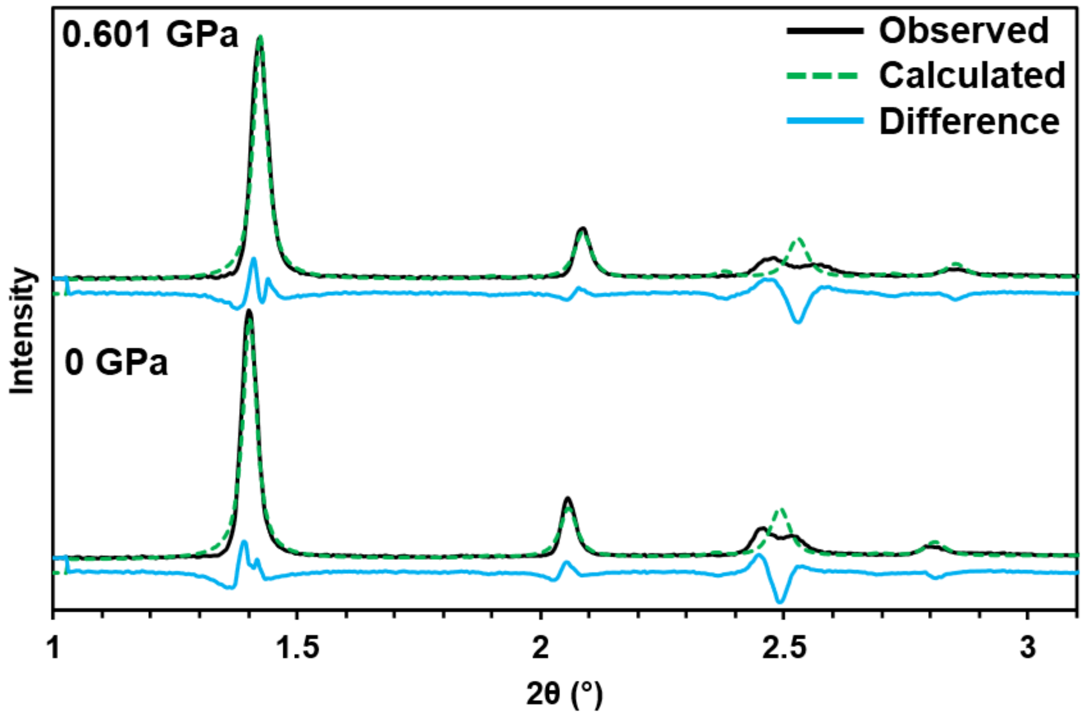

Supplementary Figure 9. Le Bail fittings of NU-1401 at 0 GPa and 0.601 GPa.

Supplementary Table 4. Parameters of Le Bail fits to variable pressure PXRD data for MIL-88B

**Pressure (GPa)**	**V CaF_2 (Å^3)**	**a MIL-88B (Å)**	**c MIL-88B (Å)**	**V MIL-88B (Å^3)**	**Rwp (%)**
0	163.307(7)	10.473(1)	19.299(1)	1833.0(4)	6.56
-0.004(4)	163.168(5)	10.472(2)	19.300(2)	1832.4(4)	7.30
0.001(4)	163.167(6)	10.470(2)	19.300(2)	1831.4(4)	7.08
0.011(4)	163.158(6)	10.467(2)	19.302(2)	1828.8(5)	7.16
0.051(4)	163.139(6)	10.459(2)	19.305(2)	1824.3(5)	7.10
0.072(4)	163.061(6)	10.445(2)	19.308(2)	1820.7(5)	6.95
0.084(4)	163.022(5)	10.434(2)	19.310(2)	1817.9(4)	6.85
0.103(4)	162.998(5)	10.425(2)	19.312(2)	1813.4(4)	6.75
0.115(4)	162.961(6)	10.412(2)	19.315(2)	1807.7(4)	6.65
0.137(4)	162.938(6)	10.395(2)	19.318(2)	1803.0(5)	6.66
0.175(4)	162.894(5)	10.381(2)	19.321(2)	1795.9(5)	6.78
0.208(4)	162.821(6)	10.359(2)	19.326(2)	1786.8(5)	6.85
0.256(4)	162.758(7)	10.331(2)	19.329(2)	1773.6(5)	6.79
0.306(4)	162.666(7)	10.292(2)	19.334(2)	1764.9(5)	6.63
0.356(4)	162.568(6)	10.266(2)	19.337(2)	1754.9(5)	6.61
0.414(4)	162.472(6)	10.236(2)	19.339(1)	1746.4(5)	6.51
0.444(4)	162.361(6)	10.211(2)	19.341(1)	1740.4(5)	6.54
0.462(4)	162.303(6)	10.194(2)	19.340(2)	1735.9(5)	6.55
0.484(4)	162.269(6)	10.180(2)	19.340(2)	1732.0(5)	6.55
0.513(4)	162.226(6)	10.169(2)	19.339(3)	1728.8(5)	6.52

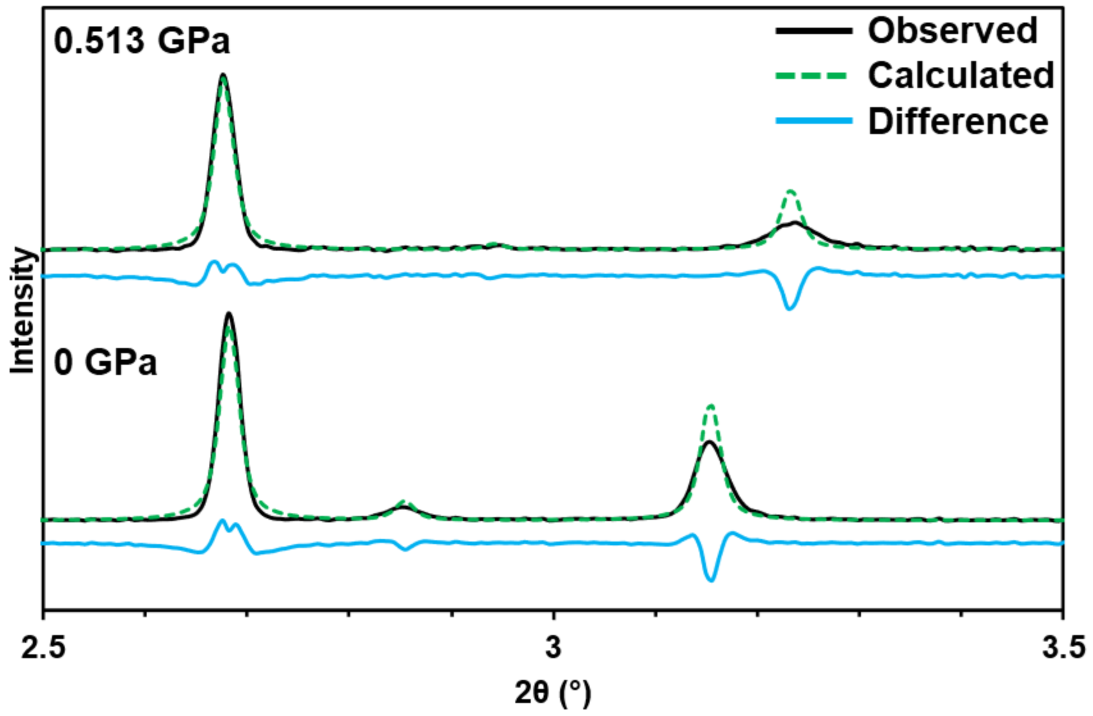

Supplementary Figure 10. Le Bail fittings of MIL-88B at 0 GPa and 0.513 GPa.

Supplementary Table 5. Parameters of Le Bail fits to variable pressure PXRD data for MIL-88B-$(CH_3)_2$

**Pressure (GPa)**	**V CaF_2 (Å^3)**	**a MIL-88B-$(CH_3)_2$ (Å)**	**c MIL-88B-$(CH_3)_2$ (Å)**	**V MIL-88B-$(CH_3)_2$ (Å^3)**	**Rwp (%)**
0	163.363(10)	10.947(13)	19.138(32)	1986(3)	5.76
0.002(7)	163.352(9)	10.943(13)	19.139(33)	1985(3)	5.30
0.029(9)	163.298(14)	10.921(13)	19.152(31)	1978(3)	5.16
0.042(9)	163.274(14)	10.900(13)	19.165(30)	1972(3)	5.30
0.066(7)	163.227(10)	10.89(13)	19.169(29)	1969(3)	5.30
0.071(7)	163.218(9)	10.882(13)	19.174(29)	1966(3)	5.32
0.075(7)	163.209(10)	10.877(13)	19.184(27)	1966(3)	5.24
0.080(7)	163.200(11)	10.872(13)	19.188(27)	1964(3)	5.38
0.098(7)	163.165(10)	10.867(13)	19.185(27)	1962(3)	5.35
0.112(8)	163.138(11)	10.849(13)	19.202(26)	1957(3)	5.36
0.112(7)	163.139(10)	10.833(13)	19.211(25)	1952(3)	5.38
0.118(7)	163.125(10)	10.818(13)	19.211(24)	1947(3)	5.47
0.158(7)	163.048(11)	10.798(12)	19.221(23)	1941(3)	5.35
0.163(7)	163.039(10)	10.784(12)	19.222(21)	1936(3)	5.43
0.196(7)	162.975(10)	10.764(12)	19.233(20)	1930(3)	5.28
0.235(7)	162.899(9)	10.739(11)	19.222(17)	1920(3)	4.62
0.263(7)	162.846(9)	10.708(11)	19.230(16)	1910(2)	4.29
0.304(7)	162.766(9)	10.669(12)	19.240(15)	1897(3)	4.56
0.338(7)	162.701(8)	10.632(13)	19.217(16)	1881(3)	4.79

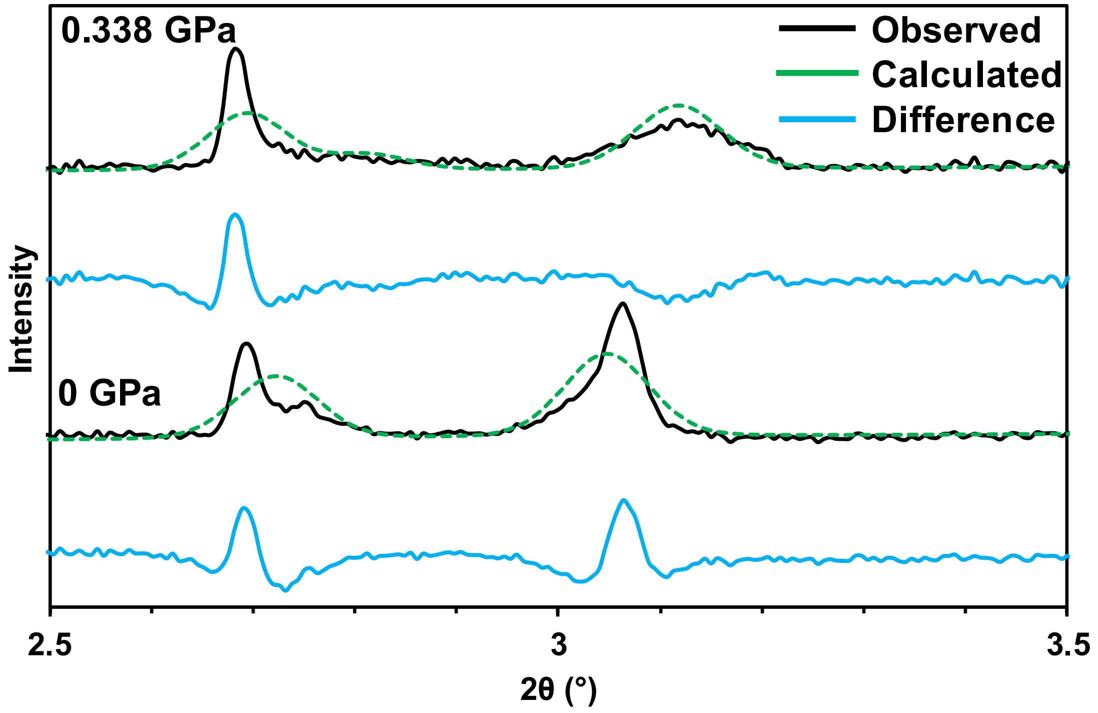

Supplementary Figure 11. Le Bail fittings of MIL-88B-$(CH_3)_2$ at 0 GPa and 0.338 GPa.

Supplementary Table 6. Parameters of Le Bail fits to variable pressure PXRD data for MIL-88B-$(CH_3)_4$

Pressure (GPa)	V CaF_2 (Å^3)	a MIL-88B-$(CH_3)_4$ (Å)	c MIL-88B-$(CH_3)_4$ (Å)	V MIL-88B-$(CH_3)_4$ (Å^3)	Rwp (%)
0	163.269	15.129(13)	16.598(14)	3290(1)	8.76
0.013(3)	163.236(7)	15.135(10)	16.575(11)	3288(1)	5.94
0.039(3)	163.186(7)	15.083(10)	16.618(11)	3274(1)	5.79
0.054(4)	163.157(7)	15.021(10)	16.680(11)	3259(1)	5.64
0.082(3)	163.103(7)	14.967(10)	16.721(11)	3244(1)	5.56
0.112(3)	163.044(7)	14.901(9)	16.779(10)	3226(1)	5.32
0.148(3)	162.975(6)	14.846(9)	16.819(10)	3210(1)	5.31
0.167(4)	162.937(7)	14.776(9)	16.883(10)	3192(1)	5.15
0.204(3)	162.866(6)	14.705(9)	16.944(10)	3173(1)	4.99
0.232(3)	162.812(6)	14.653(9)	16.987(10)	3159(1)	4.46
0.255(4)	162.769(7)	14.620(8)	17.014(10)	3149(1)	4.20
0.258(4)	162.763(7)	14.621(8)	17.003(10)	3148(1)	4.03

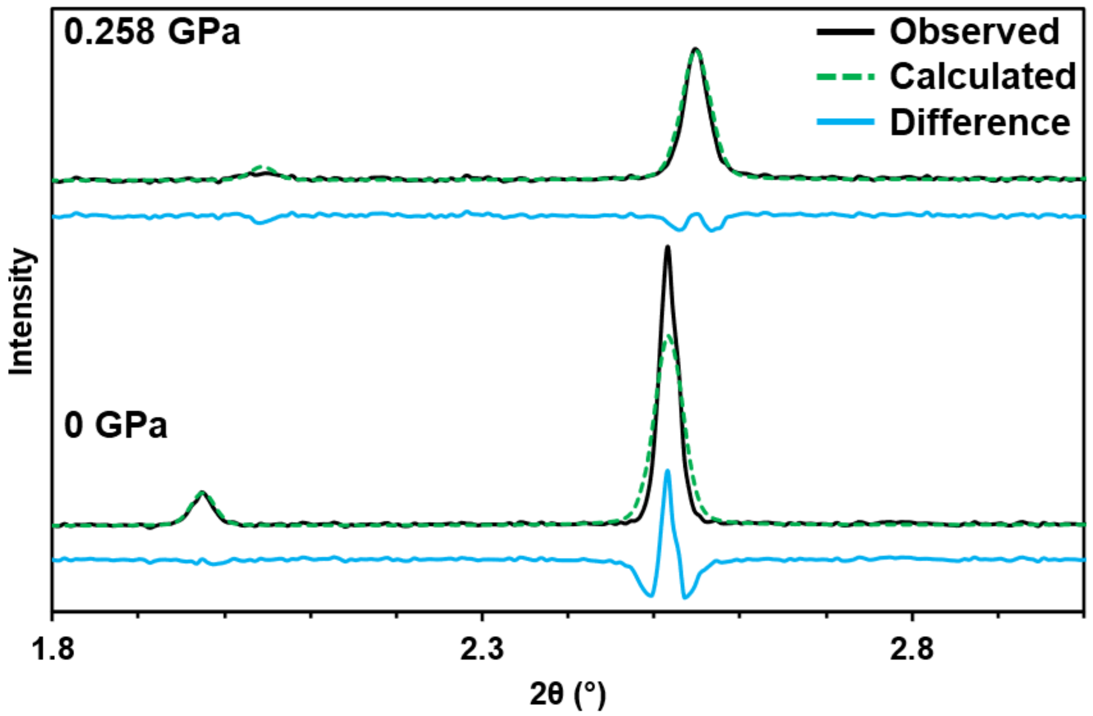

Supplementary Figure 12. Le Bail fittings of MIL-88B-$(CH_3)_4$ at 0 GPa and 0.258 GPa.

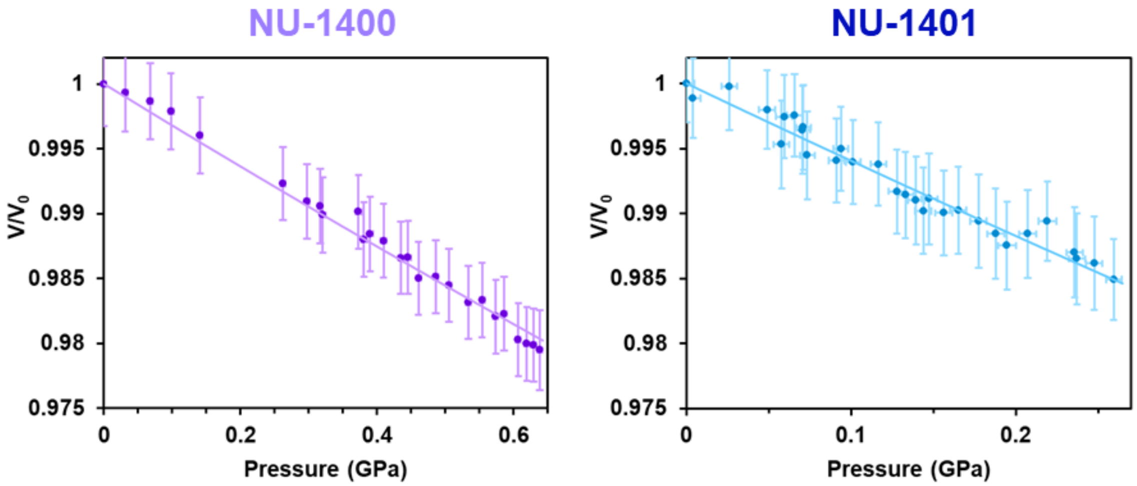

Supplementary Figure 13. Additional pressurization trials for NU-1400 (*K* = 30.9 ± 0.3 GPa) and NU-1401 (*K* = 16.5 ± 0.3 GPa). Data was collected at the Advanced Photon Source, Argonne National Laboratory using λ = 0.45194 Å for NU-1400 and λ = 0.452564 Å for NU-1401. A different sample batch was measured for NU-1401 compared to the sample analyzed in the main text. Error bars represent estimated standard deviations of fittings.

Supplementary Table 7. Parameters of Le Bail fits to variable pressure PXRD data for NU-1400, second trial

Pressure (GPa)	V CaF_2 (Å^3)	a NU-1400(Å)	b NU-1400(Å)	c NU-1400 (Å)	V NU-1400 (Å^3)	Rwp (%)
0	163.191(5)	25.204(8)	29.862(11)	9.015(20)	6785(16)	15.82
0.031(3)	163.123(5)	25.179(8)	29.857(10)	9.019(17)	6780(13)	15.34
0.068(3)	163.052(4)	25.165(8)	29.849(9)	9.020(16)	6776(12)	14.80
0.098(3)	162.993(4)	25.157(8)	29.828(9)	9.023(16)	6771(12)	14.05
0.141(3)	162.910(4)	25.142(8)	29.796(9)	9.021(16)	6758(12)	13.76
0.262(4)	162.676(5)	25.108(9)	29.724(9)	9.021(15)	6733(11)	14.20
0.298(3)	162.608(4)	25.101(8)	29.689(9)	9.022(16)	6724(12)	14.42
0.317(3)	162.571(4)	25.095(8)	29.678(9)	9.024(16)	6721(12)	14.34
0.321(4)	162.564(5)	25.091(8)	29.666(9)	9.023(16)	6716(12)	14.26
0.330(4)	162.546(4)	25.090(8)	29.655(9)	9.035(16)	6722(12)	14.27
0.373(4)	162.463(5)	25.076(8)	29.621(9)	9.044(15)	6718(11)	14.11
0.381(4)	162.448(6)	25.071(8)	29.609(9)	9.031(15)	6704(12)	14.12
0.390(4)	162.431(6)	25.072(8)	29.594(9)	9.039(16)	6706(12)	13.97
0.410(4)	162.393(6)	25.065(8)	29.580(9)	9.041(15)	6703(12)	13.75
0.435(4)	162.344(6)	25.059(8)	29.562(9)	9.036(15)	6694(11)	13.55
0.446(4)	162.323(6)	25.058(8)	29.546(9)	9.042(15)	6694(11)	13.51
0.461(4)	162.294(5)	25.050(8)	29.525(9)	9.036(15)	6683(11)	13.43
0.486(3)	162.246(5)	25.043(8)	29.505(9)	9.046(15)	6684(11)	13.63
0.505(4)	162.209(5)	25.036(8)	29.486(9)	9.049(15)	6680(11)	13.66
0.534(4)	162.154(5)	25.030(8)	29.464(9)	9.045(15)	6671(11)	13.76
0.554(4)	162.116(5)	25.022(8)	29.446(9)	9.055(16)	6672(12)	14.09
0.574(3)	162.078(5)	25.015(8)	29.432(9)	9.051(16)	6663(12)	14.38
0.586(3)	162.054(4)	25.012(8)	29.420(9)	9.057(16)	6665(12)	14.34
0.607(3)	162.015(4)	25.007(8)	29.403(9)	9.046(15)	6651(12)	14.46
0.619(3)	161.991(4)	25.001(8)	29.389(9)	9.049(15)	6649(12)	14.37
0.629(3)	161.972(4)	24.998(8)	29.377(9)	9.054(15)	6648(12)	14.49

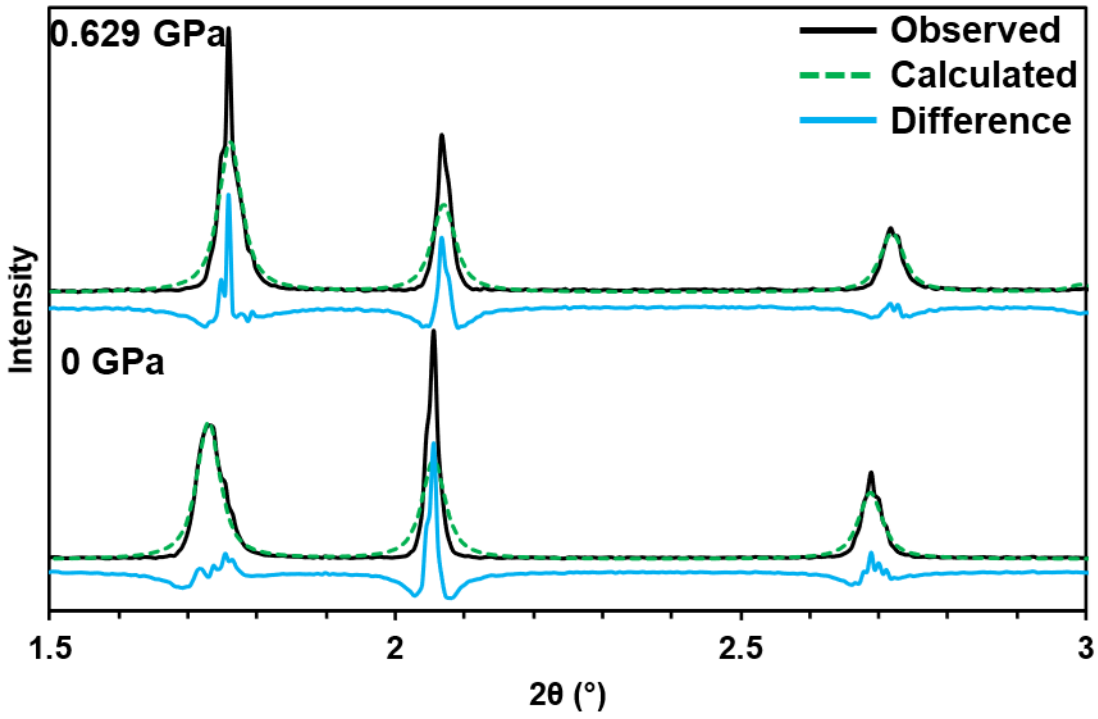

Supplementary Figure 14. Le Bail fittings of NU-1400 at 0 GPa and 0.629 GPa.

Supplementary Table 8. Parameters of Le Bail fits to variable pressure PXRD data for NU-1401, second trial

**Pressure (GPa)**	**V CaF_2 (Å^3)**	**a NU-1400(Å)**	**b NU-1400(Å)**	**c NU-1400 (Å)**	**V NU-1400 (Å^3)**	**Rwp (%)**
0	163.276(6)	16.332(34)	25.312(9)	36.286(5)	15001(32)	11.37
0.004(3)	163.260(7)	16.319(35)	25.306(9)	36.283(5)	14984(33)	11.14
0.026(3)	163.217(6)	16.342(42)	25.301(9)	36.273(5)	14998(39)	11.27
0.049(3)	163.173(7)	16.323(35)	25.296(9)	36.258(5)	14971(32)	11.10
0.059(3)	163.153(7)	16.325(38)	25.288(10)	36.244(5)	14963(36)	11.27
0.066(3)	163.141(6)	16.332(38)	25.286(10)	36.236(5)	14964(35)	11.29
0.071(3)	163.131(7)	16.322(43)	25.277(9)	36.230(5)	14947(40)	11.23
0.071(3)	163.130(6)	16.329(40)	25.278(10)	36.220(5)	14950(37)	11.19
0.058(3)	163.156(6)	16.313(42)	25.273(10)	36.214(5)	14931(39)	11.28
0.073(3)	163.126(6)	16.304(43)	25.272(10)	36.206(5)	14918(40)	11.22
0.091(3)	163.091(6)	16.306(39)	25.264(10)	36.198(5)	14912(36)	11.06
0.101(3)	163.072(6)	16.311(40)	25.260(10)	36.189(5)	14910(37)	11.30
0.094(3)	163.086(6)	16.331(40)	25.260(10)	36.182(5)	14926(37)	11.15
0.116(3)	163.0420(6)	16.322(39)	25.251(10)	36.171(5)	14908(36)	11.20
0.128(3)	163.020(6)	16.298(39)	25.244(10)	36.158(5)	14877(36)	11.16
0.133(3)	163.010(7)	16.300(41)	25.241(10)	36.149(5)	14872(38)	11.31
0.139(3)	162.998(7)	16.294(43)	25.241(10)	36.145(5)	14866(40)	11.31
0.144(3)	162.989(7)	16.287(42)	25.237(10)	36.138(5)	14854(39)	11.24
0.147(4)	162.982(7)	16.305(46)	25.237(10)	36.131(5)	14868(42)	11.36
0.156(3)	162.965(7)	16.296(41)	25.230(10)	36.122(5)	14852(38)	11.35
0.165(3)	162.948(7)	16.305(42)	25.226(10)	36.117(5)	14855(39)	11.39
0.177(4)	162.924(7)	16.294(47)	25.226(10)	36.109(6)	14842(44)	11.57
0.188(3)	162.904(7)	16.286(45)	25.219(10)	36.101(5)	14828(42)	11.44
0.195(3)	162.891(7)	16.275(43)	25.219(10)	36.093(5)	14814(40)	11.53
0.207(3)	162.867(8)	16.296(43)	25.214(10)	36.086(5)	14827(39)	11.64
0.219(3)	162.843(7)	16.319(35)	25.210(10)	36.076(6)	14842(33)	12.00

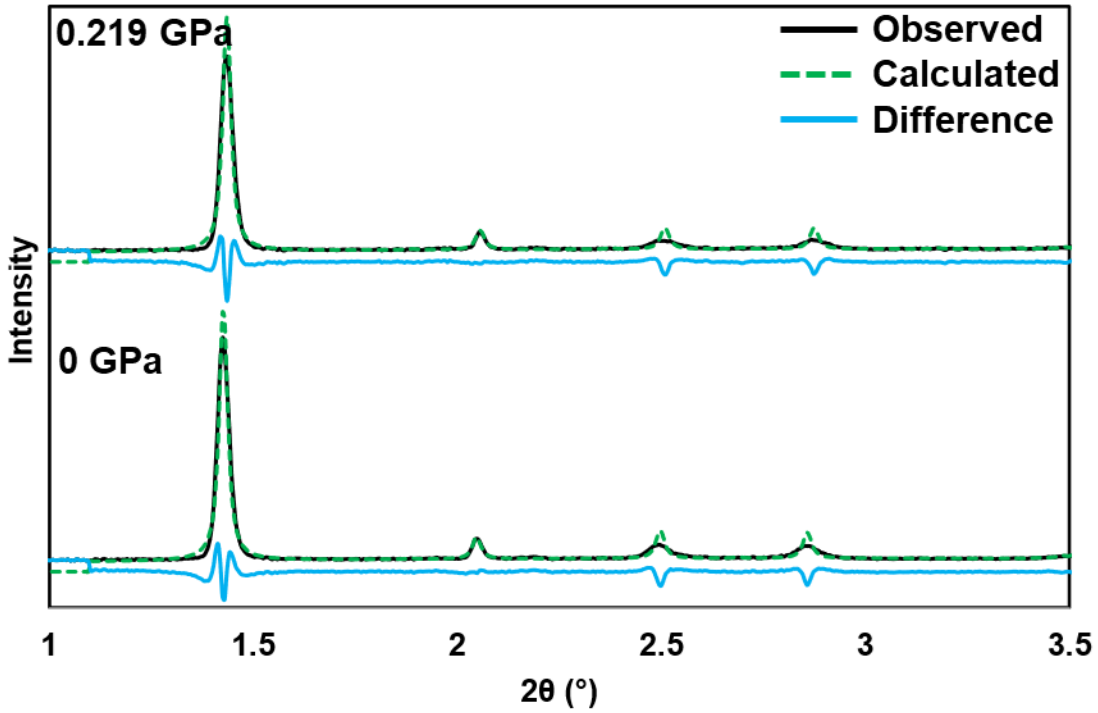

Supplementary Figure 15. Le Bail fittings of NU-1401 at 0 GPa and 0.219 GPa.

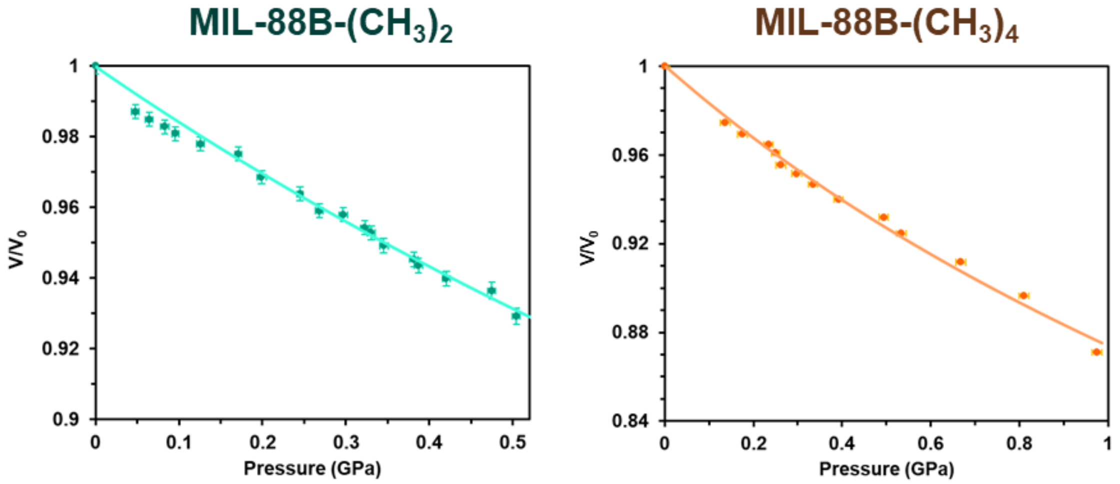

Supplementary Figure 16. Additional pressurization trials for MIL-88B-$(CH_3)_2$ (K = 6.1 ± 0.1 GPa) and MIL-88B-$(CH_3)_4$ (K = 5.7 ± 0.1 GPa). Data was collected at the Advanced Photon Source, Argonne National Laboratory using λ = 0.45194 Å for MIL-88B-$(CH_3)_2$ and λ = 0.452564 Å for MIL-88B-$(CH_3)_4$. A different sample batch was measured for MIL-88B-$(CH_3)_4$ compared to the sample analyzed in the main text. Error bars represent estimated standard deviations of fittings.

Supplementary Table 9. Parameters of Le Bail fits to variable pressure PXRD data for MIL-88B-$(CH_3)_2$, second trial

**Pressure (GPa)**	**V CaF_2 (Å^3)**	**a MIL-88B-$(CH_3)_2$ (Å)**	**c MIL-88B-$(CH_3)_2$ (Å)**	**V MIL-88B-$(CH_3)_2$ (Å^3)**	**Rwp (%)**
0	163.280(9)	10.940(15)	19.145(33)	1984(3)	7.44
0.048(5)	163.179(9)	10.842(11)	19.241(16)	1959(3)	6.19
0.064(4)	163.147(9)	10.829(11)	19.245(15)	1954(3)	6.03
0.083(5)	163.111(9)	10.819(11)	19.238(14)	1950(2)	5.88
0.096(4)	163.086(8)	10.807(11)	19.244(13)	1946(2)	5.75
0.126(5)	163.028(9)	10.790(10)	19.246(12)	1941(2)	5.31
0.171(4)	162.940(8)	10.771(10)	19.258(12)	1935(2)	5.07
0.199(5)	162.887(10)	10.749(10)	19.209(12)	1922(2)	4.87
0.245(4)	162.798(7)	10.723(11)	19.204(12)	1912(2)	5.02
0.268(5)	162.753(9)	10.695(11)	19.212(11)	1903(2)	5.05
0.297(5)	162.648(10)	10.688(11)	19.213(12)	1901(2)	5.09
0.323(5)	162.633(9)	10.667(11)	19.214(12)	1894(2)	5.19
0.330(5)	162.605(10)	10.655(12)	19.230(12)	1891(3)	5.44
0.345(5)	162.535(10)	10.639(13)	19.211(13)	1883(3)	5.70
0.381(5)	162.524(9)	10.608(13)	19.250(12)	1876(3)	5.80
0.387(5)	162.502(9)	10.612(14)	19.195(14)	1872(3)	6.27
0.420(5)	162.460(9)	10.583(14)	19.228(13)	1865(3)	6.01
0.475(5)	162.356(9)	10.555(18)	19.257(15)	1858(4)	6.52
0.504(5)	162.299(9)	10.520(15)	19.237(13)	1844(3)	6.50

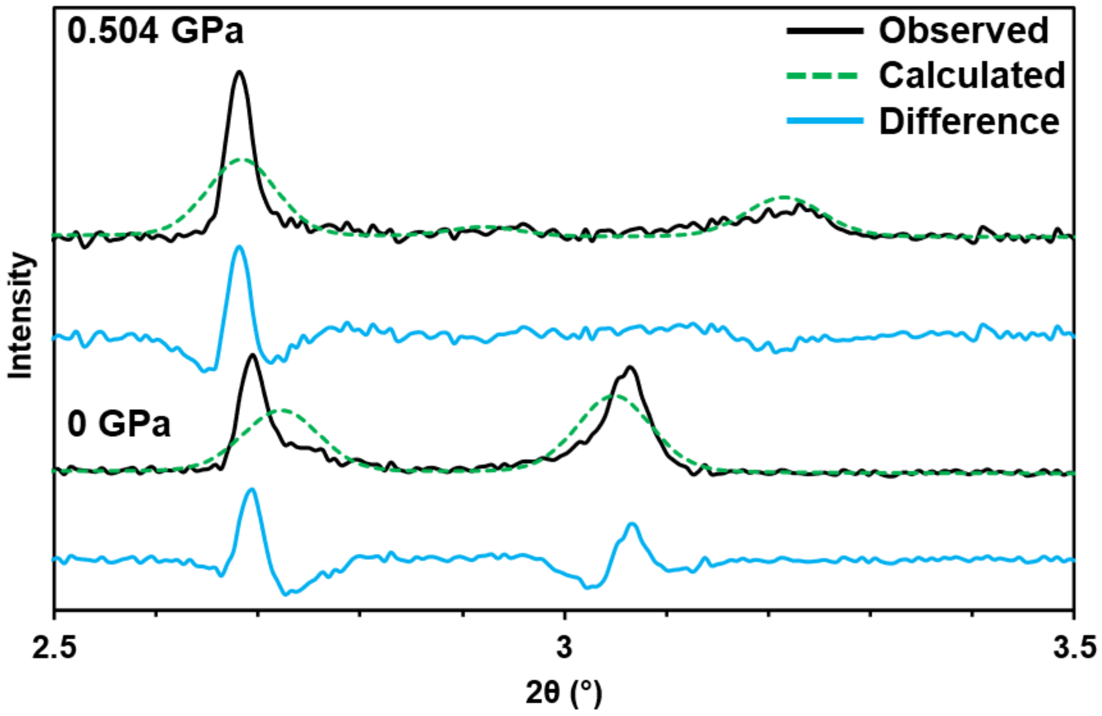

Supplementary Figure 17. Le Bail fittings of MIL-88B-$(CH_3)_2$ at 0 GPa and 0.504 GPa.

Supplementary Table 10. Parameters of Le Bail fits to variable pressure PXRD data for MIL-88B-$(CH_3)_4$, second trial

Pressure (GPa)	V CaF_2 (Å^3)	a MIL-88B-$(CH_3)_4$ (Å)	c MIL-88B-$(CH_3)_4$ (Å)	V MIL-88B-$(CH_3)_4$ (Å^3)	Rwp (%)
0	163.228(15)	14.975(4)	16.702(5)	3244(1)	4.70
0.136(11)	162.957(15)	14.758(6)	16.760(6)	3161(1)	7.30
0.175(10)	162.881(14)	14.624(5)	16.975(6)	3144(1)	6.00
0.234(10)	162.767(12)	14.579(5)	17.001(6)	3129(1)	6.04
0.25(10)	162.737(12)	14.538(5)	17.027(6)	3116(1)	6.07
0.262(11)	162.713(15)	14.476(5)	17.075(6)	3099(1)	5.97
0.297(11)	162.646(14)	14.436(6)	17.103(6)	3087(1)	6.07
0.334(10)	162.574(13)	14.393(6)	17.116(7)	3071(1)	6.21
0.392(10)	162.463(14)	14.314(6)	17.182(7)	3049(1)	6.31
0.494(10)	162.267(12)	14.251(6)	17.185(7)	3023(1)	6.60
0.534(11)	162.191(15)	14.209(7)	17.150(8)	2999(1)	7.25
0.668(11)	161.935(15)	14.156(9)	17.037(11)	2957(1)	8.49
0.810(11)	161.664(15)	14.104(12)	16.876(14)	2908(1)	10.11
0.976(11)	161.349(15)	14.043(13)	16.544(15)	2825(2)	10.14

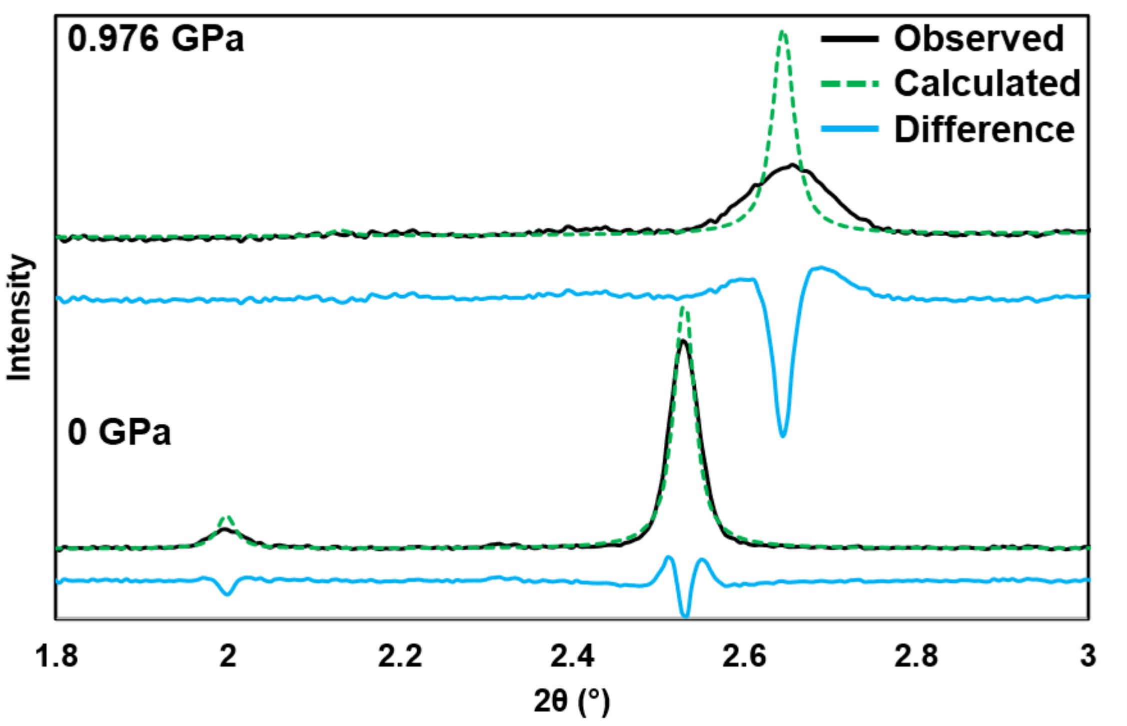

Supplementary Figure 18. Le Bail fittings of MIL-88B-(CH_3)42at 0 GPa and 0.976 GPa.

VI. Reversibility of compression measurements

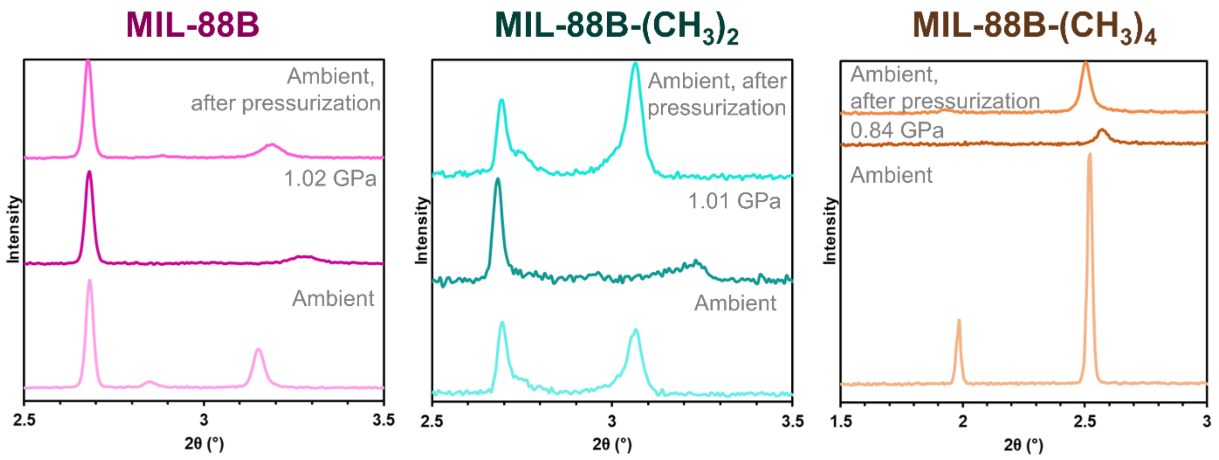

Supplementary Figure 19. Reversibility of compression after pressure campaign for MIL-88B, MIL-88B-$(CH_3)_2$, and MIL-88B-$(CH_3)_4$.

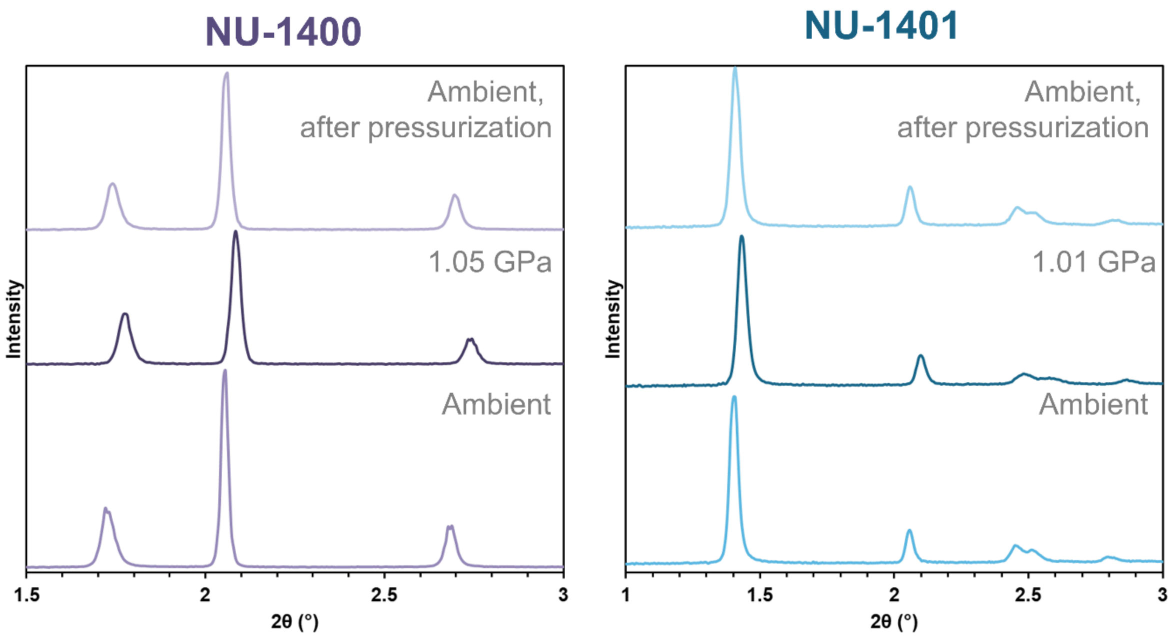

Supplementary Figure 20. Reversibility of compression after pressure campaign for NU-1400 and NU-1401.

VII. ^{1}H NMR spectrum of linker

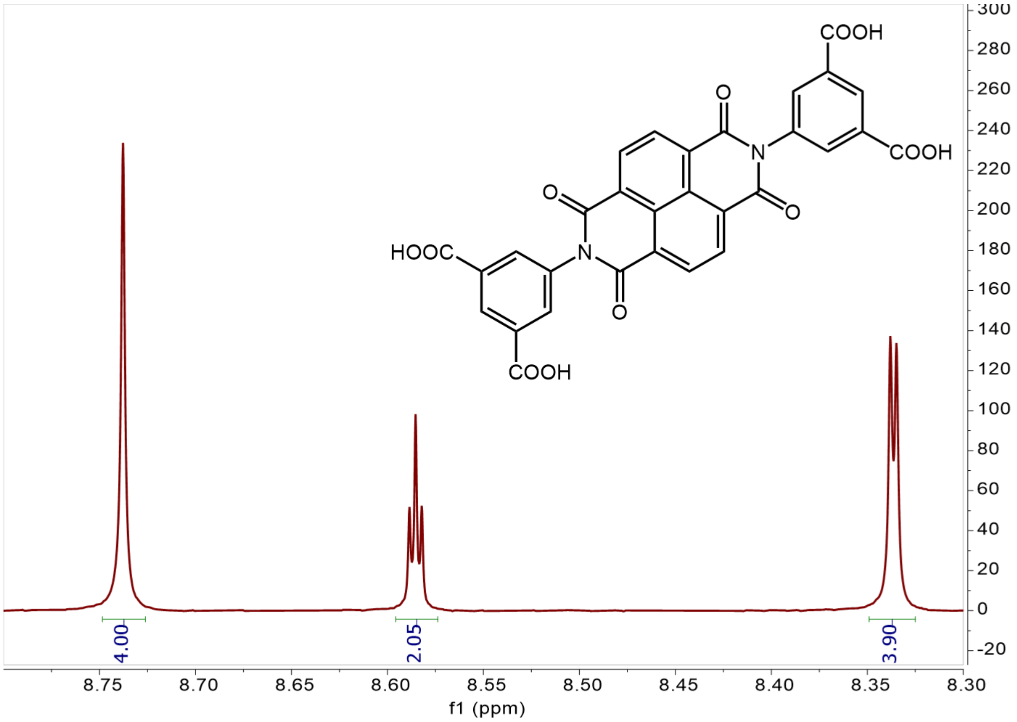

Supplementary Figure 21. ^{1}H NMR spectrum of synthesized linker
